# Joint enzyme–reaction retrieval and catalytic optima prediction via multimodal fusion

**DOI:** 10.1093/bioinformatics/btag764

**Published:** 2026-10-12

**Authors:** Yueyi Cai, Feng Yang, Juan Liu

**Affiliations:** School of Artificial Intelligence, Wuhan University, No. 299, Bayi Road, Luojia Hill Street, Wuhan, Hubei, 430072, China; School of Computer Science, Wuhan University, No. 299, Bayi Road, Luojia Hill Street, Wuhan, Hubei, 430072, China; School of Artificial Intelligence, Wuhan University, No. 299, Bayi Road, Luojia Hill Street, Wuhan, Hubei, 430072, China; School of Computer Science, Wuhan University, No. 299, Bayi Road, Luojia Hill Street, Wuhan, Hubei, 430072, China

## Abstract

**Motivation:**

Enzyme–reaction retrieval prioritizes candidate biocatalysts for experimental follow-up, where recommendations should indicate catalytic compatibility and suitable pH and temperature conditions. Existing retrieval models optimize matching scores, whereas catalytic optima predictors are typically enzyme-level regressors because condition annotations are sparse. This separation leaves ranked pairs unevaluated for condition suitability and optima predictions without reaction context.

**Results:**

We present GERO, a multimodal fusion framework integrating enzyme sequence semantics, sequence-derived pocket geometry, and molecular reaction representations through feature-gated fusion for condition-aware retrieval and catalytic optima estimation. We define the tolerance-restricted hit rate (Hit@k-TR), which requires both correct top-k retrieval and condition predictions within predefined tolerances. Across enzyme- and reaction-similarity splits, GERO improves Hit@k-TR over two-stage baselines under the primary tolerances. Benchmark examples and an iodinin biosynthesis case study illustrate candidate ranking and assay-condition estimation for experimental prioritization.

**Availability and implementation:**

Source code is available at https://github.com/ykxhs/GERO.

## 1 Introduction

Enzymes catalyze diverse biochemical transformations and are widely used in synthetic biology, pharmaceutical manufacturing and green chemistry (Kirk *et al.* 2002, Hemalatha *et al.* 2013). For computational biocatalyst discovery, a useful recommendation must answer two related questions. First, is the enzyme likely to catalyze the target reaction? Second, at what pH and temperature should the candidate be assayed? Both are important for experimental follow-up because pH and temperature strongly influence enzyme activity and stability (Fromm 1975, Almeida and Marana 2019).

Most existing computational methods focus on one of these questions. Enzyme function annotation and substrate-specificity models infer catalytic roles from sequence, structure or enzyme-substrate representations. For example, CLEAN uses contrastive protein representations for EC-number prediction (Yu *et al.* 2023), whereas EZSpecificity uses enzyme-pocket and substrate representations to model substrate specificity (Cui *et al.* 2025). More recently, enzyme–reaction retrieval has framed catalytic matching as a cross-modal ranking task. CLIPZyme (Mikhael *et al.* 2024), ReactZyme (Hua *et al.* 2024) and CREEP, adapted from CARE (Yang *et al.* 2024), rank candidate reactions for a query enzyme or candidate enzymes for a query reaction by aligning enzyme and reaction representations. These approaches address catalytic matching, but their retrieval scores do not by themselves provide pH and temperature estimates for the ranked pairs. In parallel, catalytic optima prediction has usually been formulated as an enzyme-level regression problem. EpHod predicts optimal pH from protein language-model representations (Gado *et al.* 2025), and Seq2Topt estimates temperature and pH optima from amino acid sequence features (Qiu *et al.* 2025). These models are valuable for annotating enzyme-level properties, but they are typically developed separately from enzyme–reaction retrieval and do not explicitly use the reaction being retrieved.

The observed catalytic optimum may vary with substrates, cofactors, assay protocols and reaction contexts, which can affect binding, stability and reaction energetics (Qiu *et al.* 2025). A straightforward solution is to sequentially couple a retrieval module with a property-prediction model, forming a two-stage retrieval-then-prediction pipeline. However, this strategy does not directly optimize the practical output needed by experimental users. The retrieval module is trained to rank catalytic matches without regard to condition accuracy, whereas a separate enzyme-level predictor estimates optima without conditioning on the reaction being retrieved. This two-stage design introduces a dependency between retrieval and prediction: if a relevant enzyme–reaction pair is omitted during retrieval, it cannot be considered by the downstream predictor.

In this article, we present GERO, a framework that models catalytic matching and catalytic condition prediction as a unified computational task. GERO enables bidirectional retrieval between enzymes and reactions while simultaneously estimating pH and temperature optima for retrieved candidates using the paired reaction as contextual information. For this formulation, GERO combines global evolutionary sequence information from ESM-2 (Lin *et al.* 2022), local geometric structures of predicted ligand-binding pockets derived from AlphaFold (Jumper *et al.* 2021) and identified by P2Rank (Krivák and Hoksza 2018), and molecular-level reaction representations encoded from reactants and products using Uni-Mol (Zhou *et al.* 2023). A feature-gating fusion module then constructs a joint enzyme–reaction representation for both matching-score prediction and catalytic optima estimation. To evaluate this joint task, we introduce the tolerance-restricted hit rate (Hit@k-TR), which requires the correct enzyme or reaction to appear among the top-ranked candidates and further requires predicted pH and temperature to fall within predefined tolerance ranges of the annotated optima. Benchmark evaluations show that GERO improves Hit@k-TR compared with two-stage baseline models, and an iodinin biosynthesis case study illustrates its application to enzyme prioritization and condition prediction for experimental follow-up.

## 2 Materials and methods

### 2.1 Task formulation

Given an enzyme–reaction pair (e,r), the goal is to estimate catalytic compatibility and predict catalytic pH and temperature optima conditioned on the paired reaction. GERO therefore predicts a matching score, an optimal pH estimate, and an optimal temperature estimate:


(1) $${\hat{s}}_{e,r},{\hat{p}}_{e,r},{\hat{T}}_{e,r}=F_{\Theta }(e,r)$$

where ${\hat{s}}_{e,r}$ is used to rank candidates, and ${\hat{p}}_{e,r}$ and ${\hat{T}}_{e,r}$ provide pH and temperature estimates for the same candidate pair. This formulation supports both enzyme-to-reaction retrieval (E→R), where candidate reactions are ranked for a query enzyme, and reaction-to-enzyme retrieval (R→E), where candidate enzymes are ranked for a query reaction. In both directions, each scored candidate pair is accompanied by pH and temperature estimates with reaction context.

### 2.2 Overview of GERO

The framework combines enzyme and reaction modalities (Fig. 1). For the enzyme modality, GERO uses an amino acid sequence embedding from ESM-2 to summarize global evolutionary and functional signals, and a sequence-derived pocket graph constructed from AlphaFold-predicted structures and P2Rank-predicted ligand-binding pockets to capture local geometric context. For the reaction modality, molecular participants are encoded with Uni-Mol and pooled into a reaction-level representation. These enzyme and reaction representations are projected to a shared latent dimension and fused by a feature-gating module that produces a joint enzyme–reaction representation for three prediction heads: the matching score, the optimal pH, and the optimal temperature.

**Figure 1:**
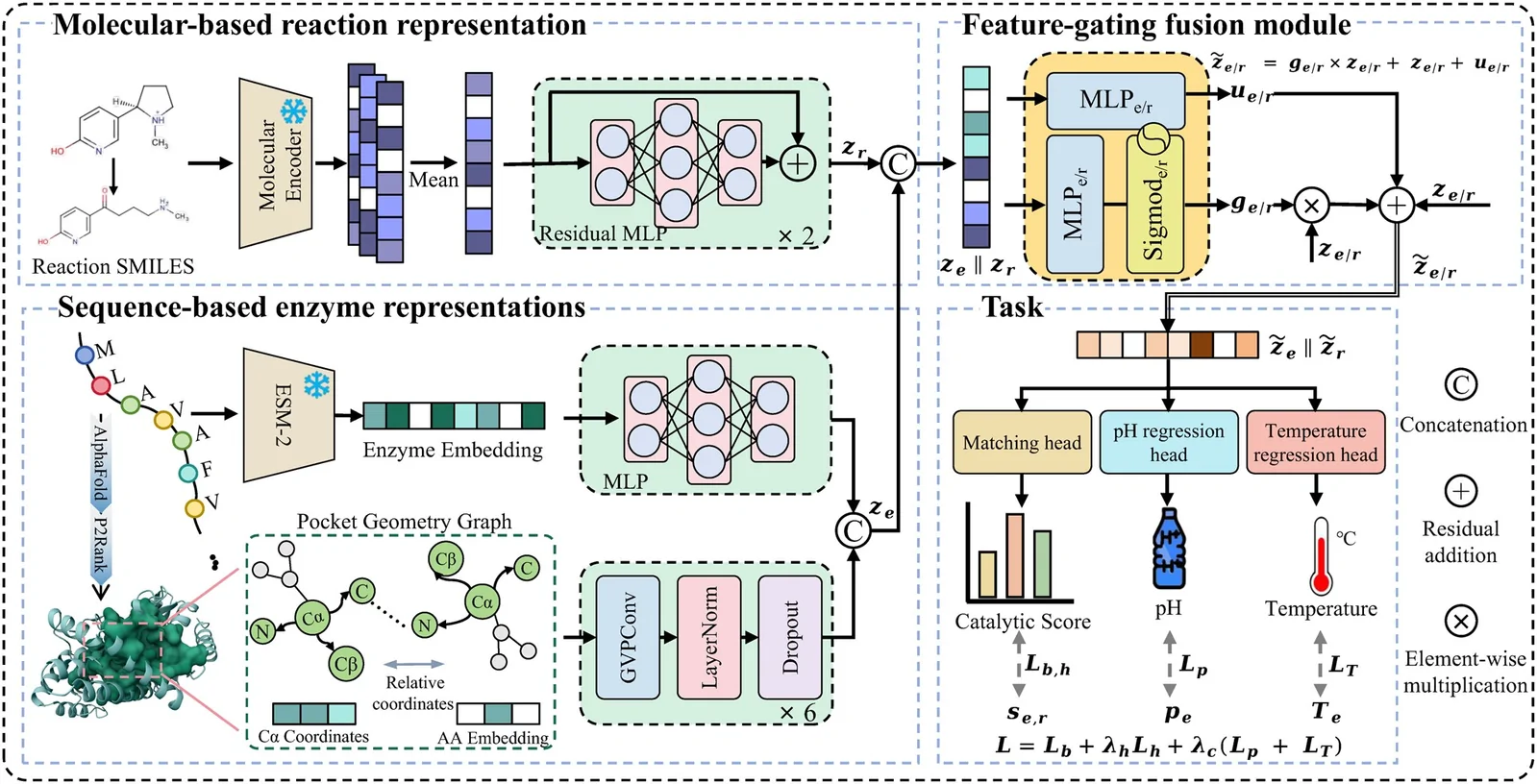
Overview of GERO. Reaction representations are fused with ESM-2 enzyme embeddings and sequence-derived pocket geometry to predict enzyme–reaction matching scores, optimal pH, and temperature.

### 2.3 Molecular-based reaction representation

Reactions are encoded from their molecular participants because the reactants and products directly define the chemical transformation that an enzyme must recognize and catalyze. GERO first encodes each molecular participant, aggregates the participant embeddings into an order-invariant reaction vector, and then projects this vector into the same latent space as the enzyme representation for cross-modal matching and optima prediction.

Let $M_{r}=\{m_{1},\ldots ,m_{N_{r}}\}$ denote the set of available molecular participants in reaction *r*, including reactants and products. Each participant is encoded by a pretrained Uni-Mol molecular encoder. The initial reaction representation $r_{\text{rxn}}$ is obtained by order-invariant mean pooling:


(2) $$r_{\text{rxn}}=\frac{1}{N_{r}}\sum_{j=1}^{N_{r}}\psi_{\text{mol}}(m_{j})$$

where $\psi_{\text{mol}}(\cdot )$ denotes the molecular encoder. This aggregation is invariant to molecular ordering and to swapping reactants and products, so it does not encode reaction direction.

The pooled reaction embedding is projected into the same latent dimension as the enzyme representation using a residual feed-forward module, $z_{r}=\phi_{r}(r_{\text{rxn}})$, where $\phi_{r}$ denotes the reaction projection network. The resulting reaction representation is used in the subsequent feature-gating fusion module.

### 2.4 Sequence-based enzyme representations

The enzyme representation is designed to capture two related but distinct aspects of retrieval: global catalytic function and local structural context. Global sequence representations capture evolutionary and functional signals that determine broad enzyme–reaction compatibility, whereas local pocket geometry provides structural context that may be relevant to substrate recognition and physicochemical constraints associated with catalytic conditions.

Given an amino acid sequence $S=(a_{1},\ldots ,a_{N_{e}})$, residue-level embeddings are obtained from a pretrained ESM-2 protein language model. The sequence representation is computed by mean pooling over non-special residues:


(3) $$e_{\text{seq}}=\frac{1}{N_{e}}\sum_{i=1}^{N_{e}}h_{i},$$

where $h_{i}$ is the final-layer contextual embedding of residue *i*. This branch provides a global summary of evolutionary and functional information.

To add local structural context without requiring experimentally resolved structures, we applied P2Rank to AlphaFold-predicted enzyme structures to identify putative ligand-binding pockets. The top-ranked predicted pocket was used to define a local pocket graph, providing a structural proxy for the putative catalytic-pocket environment. Let c be the predicted pocket center and $x_{i}$ the $C_{\alpha }$ coordinate of residue *i*. The pocket node set is $V_{e}=\{i|\|x_{i}-c{\|}_{2}\leq \rho \}$, with ρ=15 Å. Edges $E_{e}$ are defined by a *k*-nearest-neighbor graph over the selected $C_{\alpha }$ coordinates.

Each node is initialized with residue identity and local orientation vectors derived from backbone atoms and side-chain direction. The resulting pocket graph $G_{e}=(V_{e},E_{e})$ is encoded by a geometric vector perceptron network (Jing *et al.* 2020), yielding the pooled pocket embedding $e_{\text{pocket}}=\text{Pool}(\text{GVP}(G_{e}))$.

The sequence and pocket embeddings are concatenated and projected to obtain the final enzyme representation, $z_{e}=\phi_{e}(e_{\text{seq}}∥e_{\text{pocket}})$, where ∥ denotes concatenation and $\phi_{e}$ is a learnable projection network.

### 2.5 Feature-gating fusion module

After obtaining the enzyme and reaction representations, GERO applies a task-adapted dual-branch feature-gating module. Unlike simple concatenation or fusion schemes that rely on a single shared gate, this module uses the joint enzyme–reaction context to generate separate feature-wise gates for the two branches and incorporates an explicit element-wise interaction term through residual updates. Given $z_{e}$ and $z_{r}$, we first form a joint context vector:


(4) $$f_{e,r}=z_{e}∥z_{r}$$

The context vector is used to generate modality-specific gates:


(5) $$g_{e}=\sigma \left(\phi_{\text{gate}}^{e}\left(f_{e,r}\right)\right),\;g_{r}=\sigma \left(\phi_{\text{gate}}^{r}\left(f_{e,r}\right)\right)$$

Because both gates are computed from the joint enzyme–reaction context, each branch can be modulated using information from its paired counterpart while retaining its own branch-specific representation. The two gating networks have independent parameters and therefore allow the enzyme and reaction features to be modulated differently under the same enzyme–reaction context.

To further capture feature-wise compatibility, we compute interaction embeddings from the element-wise product:


(6) $$u_{e}=\phi_{\text{int}}^{e}\left(z_{e}⊙z_{r}\right),\;u_{r}=\phi_{\text{int}}^{r}\left(z_{e}⊙z_{r}\right)$$

where ⊙ denotes element-wise multiplication. The modulated enzyme and reaction representations are then computed as:


(7) $${\tilde{z}}_{e}=\text{LN}\left(z_{e}+g_{e}⊙z_{e}+u_{e}\right),\;{\tilde{z}}_{r}=\text{LN}\left(z_{r}+g_{r}⊙z_{r}+u_{r}\right)$$

where LN(·) denotes layer normalization. The final pair representation is $z_{e,r}={\tilde{z}}_{e}∥{\tilde{z}}_{r}$. Here, the gating terms control the feature-wise modulation of each branch, whereas $u_{e}$ and $u_{r}$ provide explicit pairwise interaction information. The residual formulation preserves the original branch-specific representations while incorporating context-dependent cross-modal refinement. The same joint enzyme–reaction representation is used for all prediction heads:


(8) $${\hat{s}}_{e,r}=\phi_{s}\left(z_{e,r}\right),\;{\hat{p}}_{e,r}=\phi_{p}\left(z_{e,r}\right),\;{\hat{T}}_{e,r}=\phi_{T}\left(z_{e,r}\right)$$

Here, ${\hat{s}}_{e,r}$ is used for candidate ranking, whereas ${\hat{p}}_{e,r}$ and ${\hat{T}}_{e,r}$ are used to evaluate condition-estimation accuracy under the tolerance criteria.

### 2.6 Training objective

The training objective is designed to learn a discriminative enzyme–reaction retrieval space while using catalytic-condition annotations where they are available. For a minibatch of *B* positive enzyme–reaction pairs ${\left\{(e_{i},r_{i})\right\}}_{i=1}^{B}$, basic in-batch negatives are constructed by randomly permuting the reaction indices, providing a basic contrastive signal for distinguishing observed catalytic associations from random mismatches. The basic matching loss is:


(9) $$\begin{array}{c} L_{b}=-\frac{1}{2B}\sum_{i=1}^{B}\left[\text{log}\;\sigma ({\hat{s}}_{i,i})+\text{log}\;\left(1-\sigma ({\hat{s}}_{i,\pi (i)})\right)\right] \end{array}$$

where π(·) denotes a random permutation with π(i)≠i, ${\hat{s}}_{i,j}$ is the predicted matching score between enzyme $e_{i}$ and reaction $r_{j}$, and σ(·) denotes the sigmoid function. This loss encourages observed enzyme–reaction pairs to receive high scores and randomly mismatched pairs from the same minibatch to receive low scores.

To improve discrimination among chemically or functionally similar candidates, we further use in-batch hard negative mining in both retrieval directions. For each enzyme anchor $e_{i}$, cosine similarities are computed between $z_{e_{i}}$ and all reaction embeddings in the minibatch. True positives, identical pairs, and candidate pairs with cosine similarity >0.95 are excluded to reduce the risk of selecting false negatives. From the remaining candidates, the top-*K* most similar reactions are selected as the hard negative set $H_{r}^{\left(i\right)}$, with K=10 in this study. Symmetrically, the same filtering and selection procedure is applied to each reaction anchor $r_{i}$ to obtain the hard negative enzyme set $H_{e}^{\left(i\right)}$. The hard-negative loss is:


(10) $$L_{h}=-\frac{1}{2BK}\sum_{i=1}^{B}\left[\sum_{r_{j}\in H_{r}^{\left(i\right)}}\;\text{log}\;\left(1-\sigma ({\hat{s}}_{i,j})\right)+\sum_{e_{j}\in H_{e}^{\left(i\right)}}\;\text{log}\;\left(1-\sigma ({\hat{s}}_{j,i})\right)\right]$$

Because optimal pH and optimal temperature annotations are incompletely reported in public databases, the two condition losses are computed using independent label masks. Samples lacking a label for a given condition are excluded only from the corresponding regression loss and remain available for retrieval training. The condition targets are enzyme-level optima, whereas the paired reaction is used as contextual information for prediction. Let $B_{p}$ and $B_{T}$ denote the labeled positive subsets for optimal pH and temperature, respectively. The condition losses are:


(11) $$\begin{array}{c} L_{p}=\frac{1}{\left|B_{p}\right|}\sum_{(e,r)\in B_{p}}{\left({\hat{p}}_{e,r}-p_{e}\right)}^{2},\\ L_{T}=\frac{1}{\left|B_{T}\right|}\sum_{(e,r)\in B_{T}}{\left({\hat{T}}_{e,r}-T_{e}\right)}^{2} \end{array}$$

If a minibatch contains no valid annotation for one condition, the corresponding loss term is omitted. The total objective is:


(12) $$L=L_{b}+\lambda_{h}L_{h}+\lambda_{c}\left(L_{p}+L_{T}\right)$$

where $\lambda_{h}$ and $\lambda_{c}$ control the relative contributions of hard-negative learning and condition prediction. In practice, condition supervision is introduced after an initial retrieval-alignment stage, allowing the model to first learn a stable enzyme–reaction matching space before optimizing catalytic optima estimates with reaction context.

## 3 Experiments and results

### 3.1 Experimental setup

#### 3.1.1 Dataset construction

We linked reviewed enzyme sequences from Swiss-Prot (UniProt Consortium 2019) to reaction annotations from Rhea (Bansal *et al.* 2022), structures from AlphaFoldDB (Jumper *et al.* 2021), and pH and temperature annotations from BRENDA (Placzek *et al.* 2017) to construct benchmark datasets. After preprocessing, the benchmark comprised 152 379 enzyme–reaction pairs involving 109 254 enzymes and 7572 reactions; 2851 enzymes had pH annotations, and 2434 had temperature annotations. The species distribution for the 10 most frequently condition-annotated species is shown in Fig. S1, available as supplementary data at *Bioinformatics* online. The Enzyme-SIM and Reaction-SIM splits were constructed using enzyme-sequence similarity clusters and ECFP6 reaction-fingerprint similarity clusters, respectively, with each cluster assigned exclusively to either the training or test set (Section S1, available as supplementary data at *Bioinformatics* online).

#### 3.1.2 Evaluation settings

We assessed retrieval using mean reciprocal rank (MRR) and Hit@k, and condition prediction using mean absolute error (MAE) and root mean squared error (RMSE). Hit@k-TR required a correct top-*k* match with pH and temperature errors within ±0.5 units and ±5°C, respectively. Joint evaluation used queries with at least one eligible positive pair bearing both labels. For Reaction-SIM, pairs involving enzymes with either condition annotated in training were excluded from condition evaluation, while candidate pools were unchanged. Metric definitions and query counts are provided in Section S2 and Table S2, available as supplementary data at *Bioinformatics* online; tolerance sensitivity is reported in Tables S3 and S4, available as supplementary data at *Bioinformatics* online.

#### 3.1.3 Baseline methods

Two-stage baselines combined SimSearch, ReactZyme (Hua *et al.* 2024) or CREEP adapted from CARE (Yang *et al.* 2024) with Seq2pH/Topt condition predictions (Qiu *et al.* 2025). GERO* combined GERO’s rankings with Seq2pH/Topt, whereas GERO-Sep used the same architecture as GERO but trained its condition heads after freezing the retrieval network. Trainable methods in the main comparison were evaluated across five seeds (40–44). Implementation details are provided in Section S3, available as supplementary data at *Bioinformatics* online.

#### 3.1.4 Parameter settings

GERO was trained with AdamW for 100 epochs using a batch size of 256 and a base learning rate of $6\times 10^{-4}$. ESM-2 and Uni-Mol were frozen, with representations projected to 256 dimensions. Loss weights were $\lambda_{h}=10.0$ and $\lambda_{c}=0.75$; condition losses were activated after epoch 30. Additional settings are provided in Section S4, available as supplementary data at *Bioinformatics* online.

### 3.2 Condition-aware retrieval and catalytic optima prediction

We evaluated E→R and R→E retrieval on Enzyme-SIM and Reaction-SIM. On Enzyme-SIM, GERO achieved the highest mean Hit@1-TR and Hit@10-TR in both retrieval directions (Fig. 2). For E→R retrieval, Hit@1-TR and Hit@10-TR reached (22.40±2.12)% and (32.53±2.69)%, respectively, exceeding the strongest external baseline, SimSearch, by 5.24 and 11.47 percentage points. GERO-Sep achieved 16.00% and 24.89%, respectively. For R→E retrieval, GERO achieved a Hit@10-TR of (34.33±1.42)%, compared with 23.51% for CREEP and 23.99% for GERO-Sep. GERO* shared identical retrieval rankings with GERO (Table S5, available as supplementary data at *Bioinformatics* online), but achieved lower Hit@10-TR scores of 21.87% for E→R and 20.48% for R→E. This comparison supports the contribution of GERO’s condition predictions to its tolerance-restricted retrieval performance. As an illustrative example (seed 42), GERO ranked all four annotated reactions for enzyme Q92769 in the top four positions, including RHEA: 69 176, which was absent from the Enzyme-SIM training partition (Fig. S2A, available as supplementary data at *Bioinformatics* online).

**Figure 2:**
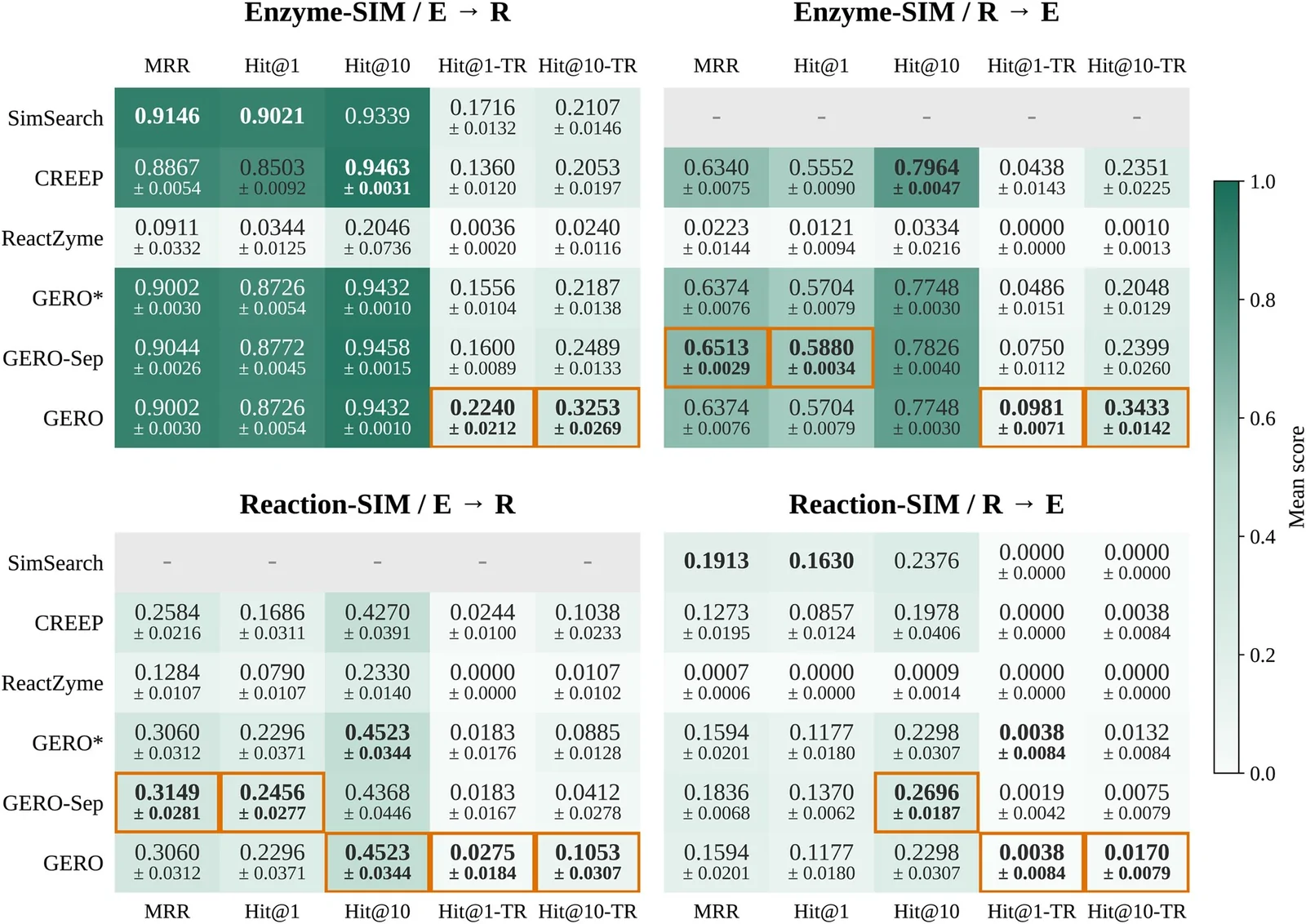
Performance heatmaps on Enzyme-SIM and Reaction-SIM for E→R and R→E retrieval (mean ± standard deviation). Bold values denote the best results across all methods within each panel. Orange boxes highlight the best results among GERO and its variants.

On Reaction-SIM, GERO’s E→R Hit@10-TR was (10.53±3.07)%, close to CREEP’s (10.38±2.33)%. For R→E retrieval, GERO achieved the highest mean Hit@10-TR of (1.70±0.79)%, compared with 0.38% for CREEP and 0.75% for GERO-Sep. GERO’s ordinary Hit@10 on the corresponding query subsets was 33.44% for E→R and 10.75% for R→E (Table S5, available as supplementary data at *Bioinformatics* online). These results highlight the difficulty of identifying correct partners that also satisfy condition constraints for reactions from held-out reaction-fingerprint clusters. Across the four retrieval settings, GERO-Sep achieved higher mean MRR and Hit@1 than GERO, whereas GERO achieved higher mean Hit@10-TR. Paired comparisons with GERO-Sep supported higher Hit@10-TR for GERO in all five seeds on Enzyme-SIM R→E, but not consistently across the other settings (Table S13, available as supplementary data at *Bioinformatics* online).

To assess the contribution of reaction context, we compared random, Top-1 retrieved, and randomly sampled positive reaction pairings on Enzyme-SIM (Fig. 3). Top-1 and Positive contexts reduced mean MAE and RMSE relative to the enzyme-only Seq2pH/Topt baseline, whereas random pairing provided no consistent improvement. For pH, MAE decreased from 0.5225 to 0.5080 with Top-1 context and 0.4964 with Positive context. For temperature, MAE decreased from 7.8897 to 7.1024 and 7.1794, respectively. Positive context yielded the lowest pH errors, while Top-1 context yielded the lowest temperature errors, supporting the value of informative reaction context for estimating enzyme-level catalytic optima. In matched controls with fixed enzyme representations and a fixed known-positive reaction context, molecular reaction representations reduced pH and temperature prediction errors relative to enzyme-only input on both splits, but did not consistently outperform EC-based context (Section S6 and Tables S6 and S7, available as supplementary data at *Bioinformatics* online). Thus, functional-class information may explain part of the benefit of reaction context. The full GERO model achieved lower regression errors than the training-mean predictor on Enzyme-SIM, whereas the training-mean predictor yielded lower errors on Reaction-SIM. Observed label distributions and predictions using retrieved Top-1 reaction context are shown in Fig. S5, available as supplementary data at *Bioinformatics* online.

**Figure 3:**
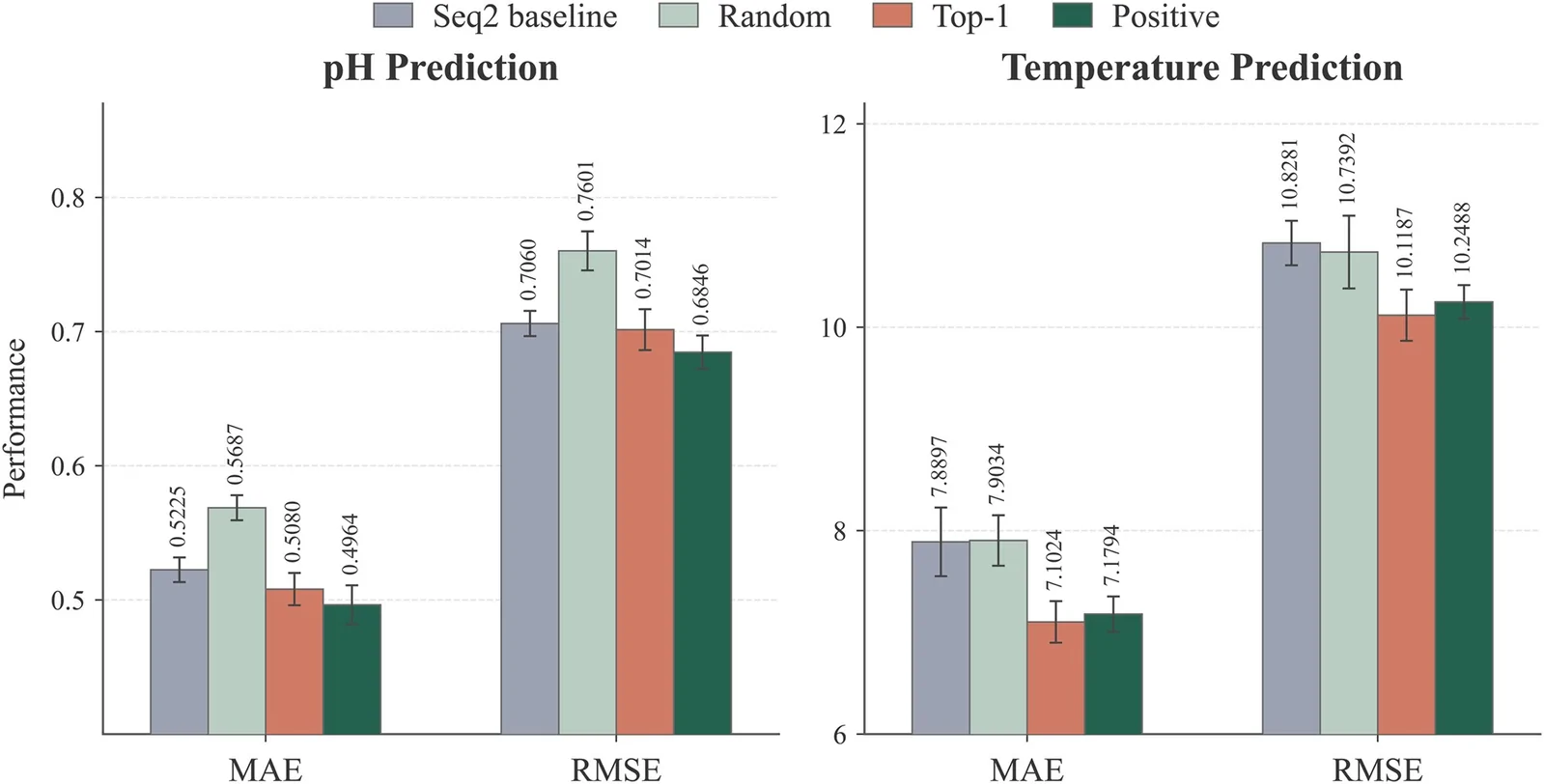
pH and temperature prediction errors on Enzyme-SIM under different reaction-context settings, compared with the enzyme-only Seq2pH/Topt baseline. Bars show mean MAE or RMSE, and error bars indicate sample standard deviations.

We further examined whether predicted catalytic optima were consistent with source-organism ecological niches on Enzyme-SIM, using known positive reaction contexts (Fig. S4 and Section S5, available as supplementary data at *Bioinformatics* online). Mean predicted optimal temperatures were higher for thermophilic than mesophilic enzymes (58.37 versus 33.76°C; Mann–Whitney *P* = 1.04 × 10^−7^), consistent with their source-organism thermal niches.

### 3.3 Analysis of module contributions and representation quality

To investigate the sources of GERO’s performance, we examined model components through seed-42 ablations across both splits and retrieval directions (Fig. 4). On Enzyme-SIM E→R, removing the pocket-derived structural branch reduced Hit@10-TR from 35.56% to 31.11%, while removing feature-gated fusion reduced it to 20.89%. Hit@1-TR also declined from 23.56% to 19.11% and 12.44%, respectively. Full retrieval metrics are provided in Tables S8 and S9, available as supplementary data at *Bioinformatics* online. Replacing Uni-Mol with RxnFP (Schwaller *et al.* 2021) or ChemBERTa (Chithrananda *et al.* 2020) reduced Enzyme-SIM E→R Hit@10-TR to 28.44% and 31.11%, respectively. However, both alternatives improved R→E Hit@10-TR, reaching 38.22% and 38.94%, compared with 32.21% for GERO. On Reaction-SIM R→E, Hit@10-TR remained below 2% across variants, with 0.94% for GERO and 1.89% without feature-gated fusion. The benefits of individual components and reaction encoders therefore depended on the benchmark split and retrieval direction.

**Figure 4:**
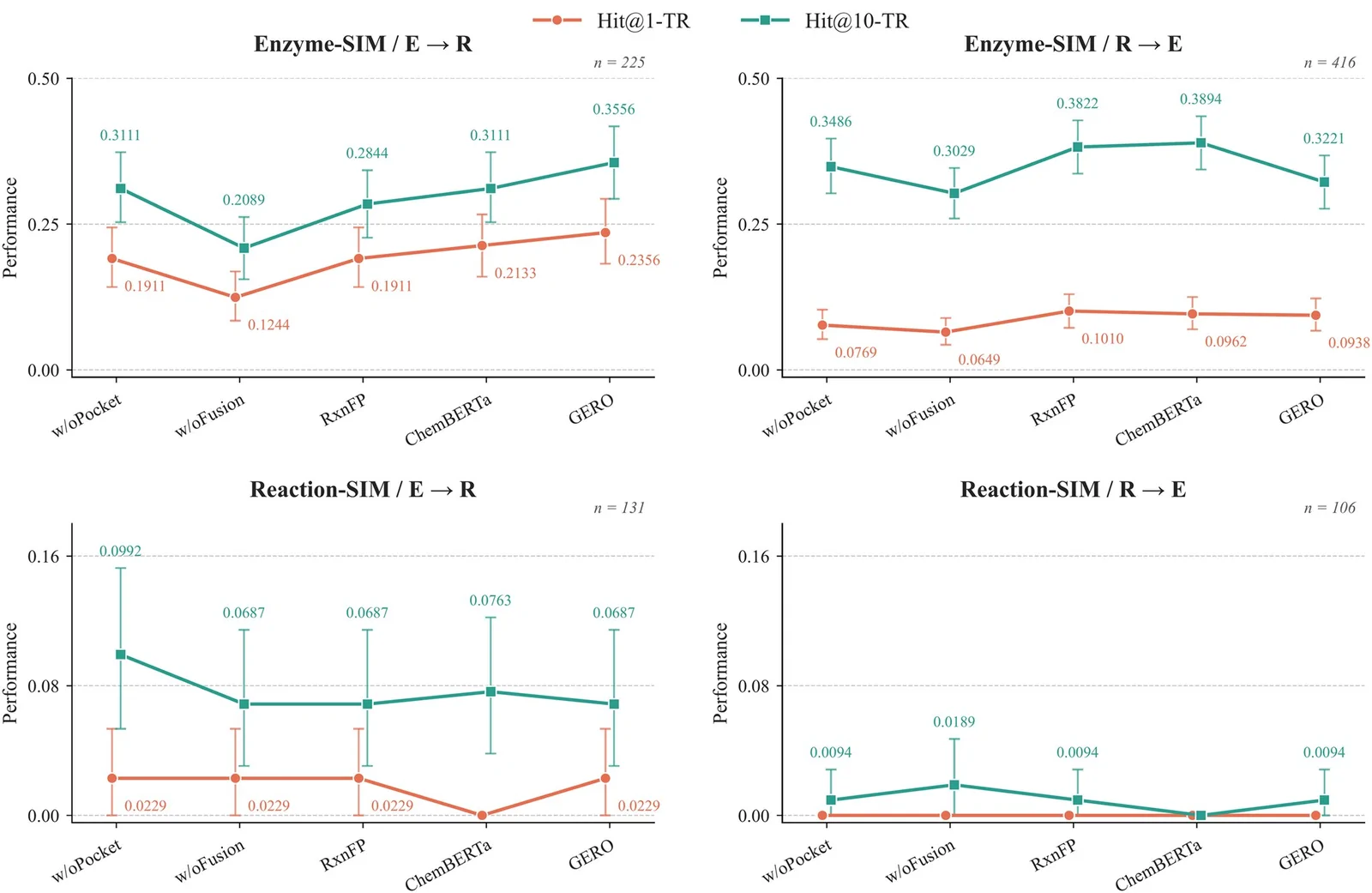
Seed-42 ablation results comparing pocket, fusion, and reaction-encoder variants across four retrieval settings. Error bars indicate pointwise 95% confidence intervals obtained from 10 000 query-level bootstrap resamples for each fitted model.

We further assessed pocket selection among enzymes with at least two valid P2Rank pockets. With model weights, cropping radius, and graph construction held fixed, the highest-scoring pocket yielded higher MRR, Hit@1, and Hit@10 than the lowest-scoring valid pocket across both splits and retrieval directions (Table S10, available as supplementary data at *Bioinformatics* online), supporting the importance of pocket selection for retrieval. Redox coenzyme exclusion was evaluated at inference using the seed-42 models, with weights and candidate pools held fixed (Table S11, available as supplementary data at *Bioinformatics* online). On Enzyme-SIM, exclusion reduced Hit@10 from 94.19% to 91.66% for E→R and from 77.26% to 72.51% for R→E, with corresponding decreases in MRR and Hit@1. Aggregate performance on Reaction-SIM remained nearly unchanged, although only two of its 644 test reactions were affected.

We further examined the learned representation through alignment-score distributions and t-SNE projections (Van der Maaten and Hinton 2008). Positive enzyme–reaction pairs generally received higher alignment scores than random negatives in both the training and test sets (Fig. S3, available as supplementary data at *Bioinformatics* online). On the Enzyme-SIM test set, t-SNE projections of fused pair representations showed local enrichment of broad EC classes, particularly EC-2 and EC-3 (Fig. 5). Coloring the projections by pH and temperature also revealed locally enriched groups. These patterns suggest that the learned representation contains information associated with enzyme function and catalytic conditions.

**Figure 5:**
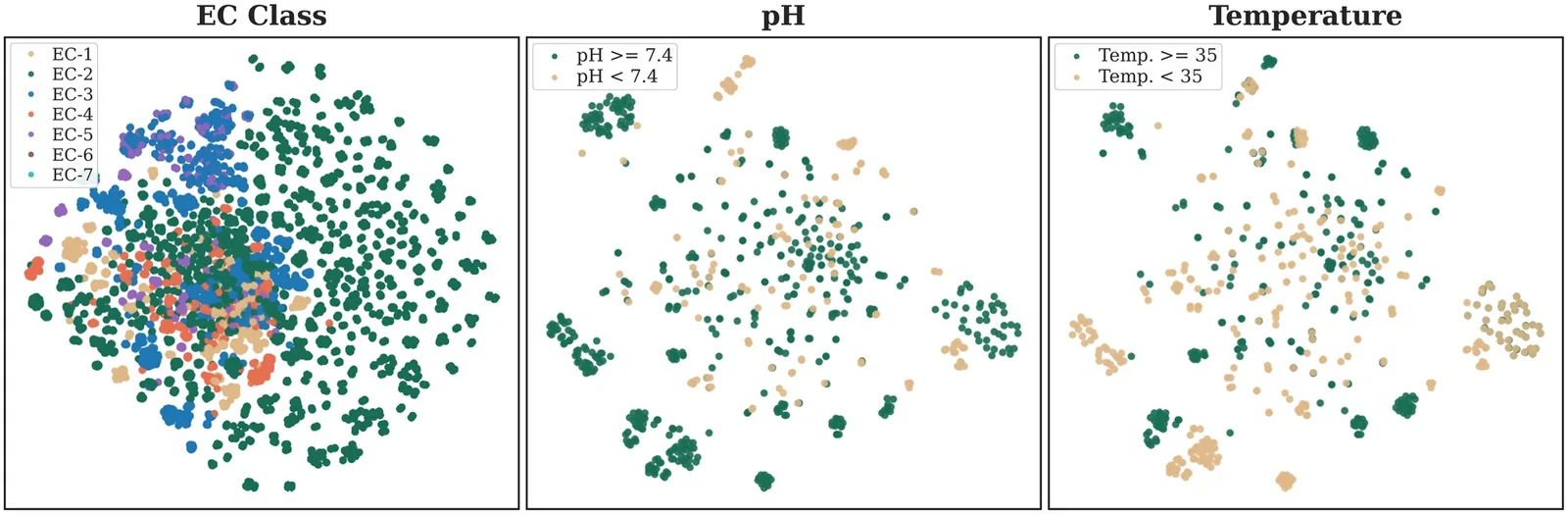
t-SNE projections of fused pair representations on the Enzyme-SIM test set, colored by EC class, pH group (threshold 7.4), and temperature group (threshold 35°C).

### 3.4 Application to iodinin biosynthesis

To examine pathway-level enzyme prioritization, we applied GERO to the iodinin biosynthesis pathway recently curated in MetaCyc (Caspi *et al.* 2018). The pathway converts phenazine-1,6-dicarboxylate to iodinin through four reactions catalyzed by xpzG, xpzH, and phzNO1 (Zhao *et al.* 2016, Shi *et al.* 2019). These enzymes and reactions were absent from the training set. We used the Reaction-SIM model with a candidate pool of 190 enzymes from relevant UniRef50 clusters with available AlphaFold structures. Reaction inputs contained the primary reactants and products, excluding coenzymes; the effects of cofactor restoration are reported in Table S12, available as supplementary data at *Bioinformatics* online.

GERO ranked xpzG sixth for both of the first two reactions, which involve larger scaffold changes, ahead of ReactZyme and CREEP (Fig. 6). For the later N-oxidation steps, ReactZyme or CREEP sometimes provided better rankings. GERO ranked xpzH 84th and 93rd among 190 candidates, limiting its usefulness for prioritizing this enzyme. Because standardized optimal pH and optimal temperature annotations were unavailable for these enzymes, we compared predictions with assay conditions reported in the literature. The xpzG/xpzH assays used pH 8.5, whereas phzNO1 assays used pH 7.5, both at ∼30°C (Shi *et al.* 2019; Zhao *et al.* 2016). Predicted pH values for xpzG/xpzH differed from the reported assay pH by 0.9–1.0 units, exceeding the ±0.5 tolerance used in this study. The reported assay conditions do not establish the enzymes’ true optima, so the predictions should be treated as initial reference points for experimental optimization.

**Figure 6:**
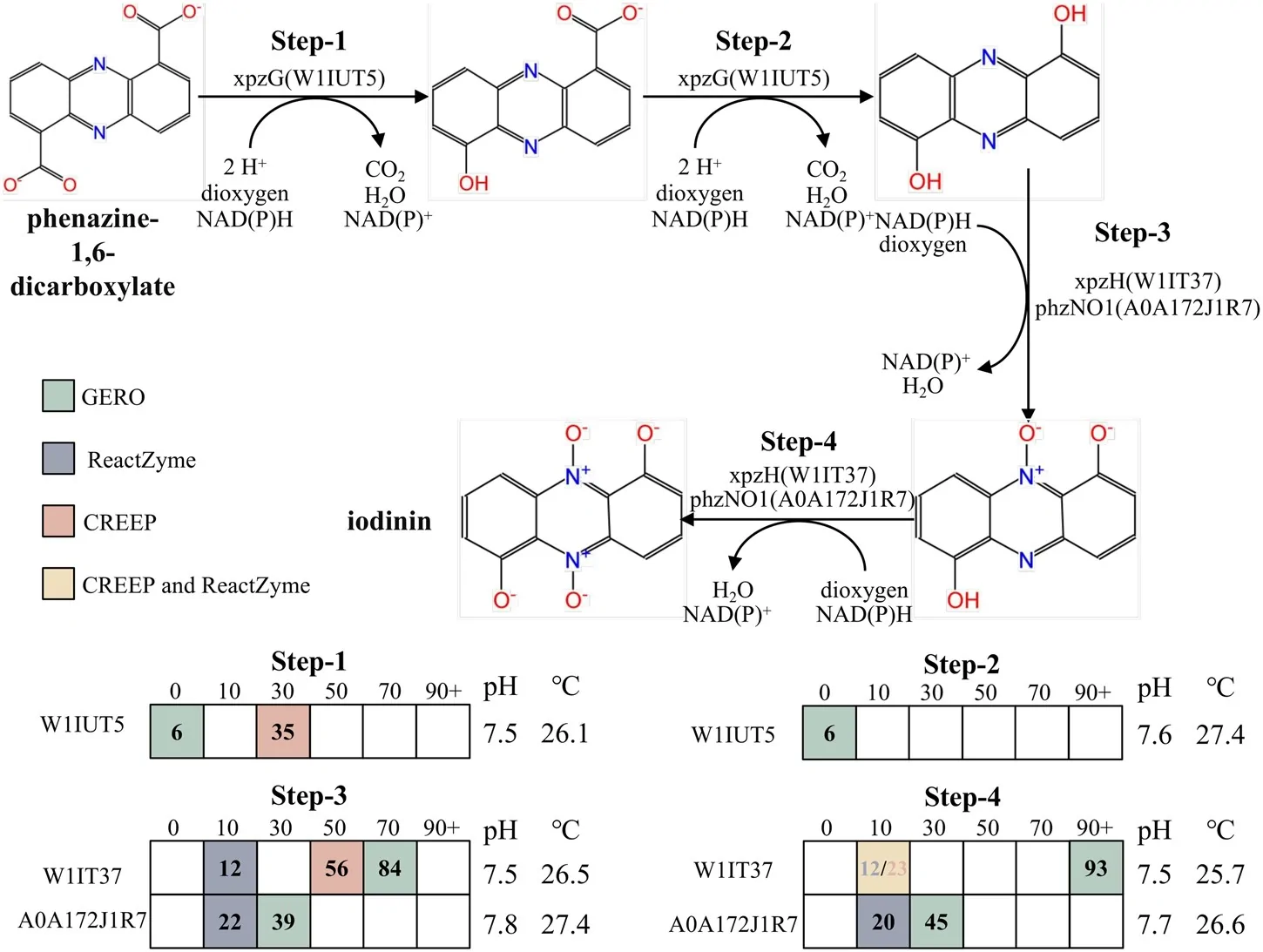
Application to iodinin biosynthesis. The pathway comprises four reaction steps catalyzed by three enzymes. The pH and temperature values estimated by GERO, together with the rankings from the comparison methods, are shown in the lower panels.

## 4 Conclusion and discussion

We presented GERO, a multimodal fusion framework for condition-aware enzyme–reaction retrieval and catalytic optima estimation with reaction context. By integrating global enzyme sequence semantics, predicted pocket geometry, and molecular reaction representations through feature-gated cross-modal fusion, GERO learns a shared enzyme–reaction representation to jointly rank candidate pairs and estimate likely optimal pH and temperature conditions.

Across both Enzyme-SIM and Reaction-SIM benchmark splits, GERO improved Hit@k-TR over two-stage retrieval-then-prediction baselines. However, several potential limitations remain. Public pH and temperature annotations are sparse and often reported at the enzyme, organism, or EC level rather than for specific enzyme–reaction pairs, preventing assessment of how optimal conditions vary across reactions catalyzed by the same enzyme. The reaction and structural representations simplify mechanistic information and rely on predicted binding pockets, which may reduce sensitivity to subtle transformations, catalytic-site changes, and mutation-induced shifts in optimal pH or temperature. In addition, the reaction-retrieval module may assign closely related wild-type and mutant sequences highly similar reaction contexts, further limiting the model’s sensitivity to mutation effects. Finally, Hit@k-TR measures agreement with database annotations rather than experimentally verified activity or screening efficiency.

Future work could extend retrieval beyond ranking within a predefined candidate space toward condition-guided enzyme candidate generation or engineering, where desired reactions and assay conditions guide the proposal of new or modified enzyme candidates. Moreover, future models could incorporate atom-mapped reaction centers, reaction-difference graphs, docking or complex-structure constraints, and uncertainty estimates. Experimental validation will ultimately be needed to determine whether condition-aware recommendations improve the efficiency of biocatalyst discovery workflows.

## Supplementary Material

btag764_Supplementary_Data

## Data Availability

Data and code supporting this study are available at https://github.com/ykxhs/GERO.
