# Supplementary material for: Joint enzyme–reaction retrieval and catalytic optima prediction via multimodal fusion
Source: Bioinformatics. 2026 Oct 12;42(10):btag764. doi: 10.1093/bioinformatics/btag764 (PMC13716482; doi:10.1093/bioinformatics/btag764)
Supplement: Supplementary file 1 [file btag764_supplementary_data.pdf]

Supplementary Materials for "Joint enzyme-reaction retrieval and catalytic optima prediction via multimodal fusion"

Yueyi Cai, Feng Yang, Juan Liu*

S1. Dataset construction and benchmark

Dataset construction: To construct a high-quality dataset of enzyme-reaction pairs alongside optimal pH and temperature, we integrated four bioinformatics databases: UniProt, Rhea, BRENDA, and AlphaFold DB. First, we retrieved all non-fragment enzyme entries with Rhea annotations from the UniProt (Swiss-Prot, release 2025.10.26) database, ensuring that the selected enzymes possessed explicit Rhea reaction annotations and were complete, manually reviewed sequences. To accommodate the maximum token length of the ESM-2 pretrained model, we filtered out enzyme entries with sequence lengths exceeding 1022. Enzyme structures for the benchmark were retrieved from AlphaFold DB v6. No pLDDT-based filtering was applied to the benchmark structures. For the iodinin biosynthesis case study, we selected the highest-confidence model generated by the AlphaFold 3 Server for each enzyme. Second, based on the retrieved Rhea identifiers, we obtained the corresponding reaction SMILES from Rhea (release 139), uniformly adopting the left-to-right direction as the standardized representation of the reactions. All reactions were subjected to impurity removal, metal disconnection, and charge neutralization. By eliminating disparities in molecular states caused by varying experimental environments, we converted them into unique and canonical representations.

Furthermore, because BRENDA (release 2025.1) organizes data by EC numbers, we iterated through all EC classes to search for entities with documented optimal pH and temperature values. For data provided as ranges, we calculated the mean. For entities with multiple data records, we discarded entries where the pH difference exceeded 1 or the temperature difference exceeded 10°C. Ultimately, the average of the remaining data was assigned as the optimal pH and temperature for the enzyme. The final constructed dataset comprises 152,379 enzyme-reaction pairs, encompassing 109,254 unique enzyme sequences and 7,572 unique chemical reactions. Among these, 2,851 enzymes possess optimal pH annotations, and 2,434 enzymes possess optimal temperature annotations. Table S1 reports the marginal availability of pH and temperature annotations.

To assess species bias in condition annotations, we grouped UniProt entries by species across strains and selected the 10 species with the most enzymes annotated for pH or temperature. These species accounted for 49.0% of pH-annotated enzymes (1,398/2,851) and 48.0% of temperature-annotated enzymes (1,168/2,434), but only 13.2% of the complete enzyme dataset (14,446/109,254; Figure S1). This imbalance indicates that supervision for condition prediction is disproportionately concentrated in a small subset of species.

Benchmark splits: We constructed two cluster-based splits to assess generalization to held-out enzyme-sequence or reaction-fingerprint clusters. For Enzyme-SIM, enzyme sequences were clustered using MMseqs2 at a 50% sequence identity threshold, with the aim of reducing potential information leakage from closely related homologs. For Reaction-SIM, each reaction was represented by summing the Morgan count fingerprints (radius = 3, corresponding to ECFP6) of its reactant and product molecules. Pairwise reaction distances were calculated as 1 - Tanimoto similarity using these aggregated count fingerprints, and reactions were clustered using the Butina algorithm with a distance threshold of 0.5. For both splits, entire clusters were assigned exclusively to either the training or test set at an approximate 9:1 ratio by cluster count, ensuring that no enzyme-sequence cluster in Enzyme-SIM or reaction-fingerprint cluster in Reaction-SIM was represented in both sets.

Queries and candidate sets: For the main benchmark, query counts and candidate-pool sizes are reported in Table S2. Enzyme-SIM used 7,572 reactions for E→R and 10,910 test enzymes for R→E; Reaction-SIM used 644 test reactions for E→R and 109,254 enzymes for R→E. Conditional joint retrieval was evaluated on queries with at least one eligible ground-truth enzyme-reaction pair annotated with both pH and temperature. For Reaction-SIM, pairs involving enzymes with either condition annotated in the training partition were excluded from this evaluation, while candidate pools remained unchanged. This filtering removes direct overlap in condition labels but does not impose sequence-disjointness between training and test enzymes. In the iodinin biosynthesis case study, the candidate pool comprised 190 enzymes with available AlphaFold structures, drawn from UniRef50 clusters associated with the target pathway enzymes. Reaction inputs included only primary

reactants and products, with coenzymes excluded to focus retrieval on the core chemical transformation.

S2. Evaluation metrics

Retrieval performance was evaluated in both enzyme-to-reaction and reaction-to-enzyme directions. For a query q, candidates from the opposite modality were ranked in descending order according to the predicted matching score $s(q,c)$. Let $\mathcal{G}(q)$ denote the set of ground-truth candidates for query q. The rank of the first correct candidate was defined as

$$\mathrm{rank}(q) = \min_{c \in \mathcal{G}(q)} \mathrm{rank}_q(c), \tag{1}$$

where $\mathrm{rank}_q(c)$ is the rank position of candidate c for query q. Hit@k was computed as

$$\mathrm{Hit@}k = \frac{1}{|Q|} \sum_{q \in Q} \mathbf{1}\left(\mathrm{rank}(q) \leq k\right), \tag{2}$$

and mean reciprocal rank (MRR) was computed as

$$\mathrm{MRR} = \frac{1}{|Q|} \sum_{q \in Q} \frac{1}{\mathrm{rank}(q)}. \tag{3}$$

The tolerance-restricted hit rate, Hit@k-TR, jointly evaluates candidate retrieval and catalytic-condition prediction. Let $P \subseteq Q$ denote the subset of queries for which at least one ground-truth enzyme-reaction pair has both pH and temperature annotations, and let $\mathcal{G}_{pt}(q) \subseteq \mathcal{G}(q)$ denote the annotated ground-truth candidates for query q. For a retrieved ground-truth candidate c, the tolerance condition was defined as

$$\mathcal{T}(q,c) = \max\left(\frac{|\hat{y}_p(q,c) - y_p(q,c)|}{\Delta_p}, \frac{|\hat{y}_T(q,c) - y_T(q,c)|}{\Delta_T} \right) \leq 1, \tag{4}$$

where $\hat{y}_p$ and $\hat{y}_T$ denote predicted optimal pH and temperature, y_p and y_T denote annotated values, and Δ_p and Δ_T denote the predefined tolerance thresholds. The set of tolerance-satisfying retrieved ground-truth candidates was

$$\mathcal{H}_k^{\mathrm{TR}}(q) = \left\{ c \in \mathrm{Top}_k(q) \cap \mathcal{G}_{pt}(q) \mid \mathcal{T}(q,c) \right\}. \tag{5}$$

Hit@k-TR was then computed as

$$\mathrm{Hit@}k\text{-TR} = \frac{1}{|P|} \sum_{q \in P} \mathbf{1}\left[\mathcal{H}_k^{\mathrm{TR}}(q) \neq \emptyset \right]. \tag{6}$$

In the primary evaluation, Δ_p was set to 0.5 pH units and Δ_T was set to 5°C. Additional sensitivity analyses used stricter and more relaxed tolerances, as reported in Tables S3 and S4.

Continuous pH and temperature prediction was evaluated using mean absolute error (MAE) and root mean squared error (RMSE) on the corresponding labeled subsets. For target $a \in \{p, T\}$ and labeled test subset $\mathcal{D}_a$, these metrics were defined as

$$\mathrm{MAE}_a = \frac{1}{|\mathcal{D}_a|} \sum_{i \in \mathcal{D}_a} \left| y_a^{(i)} - \hat{y}_a^{(i)} \right|, \tag{7}$$

$$\mathrm{RMSE}_a = \sqrt{ \frac{1}{|\mathcal{D}_a|} \sum_{i \in \mathcal{D}_a} \left(y_a^{(i)} - \hat{y}_a^{(i)} \right)^2 }. \tag{8}$$

S3. Baseline implementation details

Because no existing method jointly performs bidirectional enzyme-reaction retrieval and catalytic optima prediction, all comparison methods were implemented as two-stage baselines. Each retrieval baseline first ranked candidate enzymes or reactions, and Seq2pH/Topt was then used to provide enzyme-level optimal pH and temperature predictions for the retrieved candidates. This design mirrors the common decoupled workflow and provides a direct comparison against the training strategy used by GERO, which incorporates reaction context.

SimSearch was used as a non-parametric similarity-search baseline. For enzyme queries, MMseqs2 was used to compute amino acid sequence identity against the training set. Reactions associated with the matched training enzymes were assigned to the query, and the maximum sequence-identity score was retained when multiple homologous enzymes mapped to the same reaction. For reaction queries, each reaction was represented

by averaged ECFP6 fingerprints of its molecular participants, and Tanimoto similarity was computed against training-set reactions. Because this implementation transfers training-set associations, it can return only target entities supported by those associations. In Enzyme-SIM R→E, the candidate test enzymes are absent from the training set, whereas in Reaction-SIM E→R, the candidate test reactions are absent. Consequently, this implementation cannot assign discriminative scores to candidate targets in these two settings. SimSearch was therefore evaluated only for Enzyme-SIM E→R and Reaction-SIM R→E. A query-independent frequency-prior baseline ranked candidate reactions by the number of distinct training enzymes associated with each reaction, using the same test queries and candidate pool as the main evaluation. On Enzyme-SIM E→R, it achieved an MRR of 0.0604, Hit@1 of 3.06%, and Hit@10 of 11.00%. These results indicate that GERO's high E→R retrieval performance cannot be explained solely by the training-set reaction frequency prior.

ReactZyme was reproduced from its open-source implementation (`https://github.com/WillHua127/ReactZyme`) using the Uni-Mol+ESM+NoGNN architecture. Because the released repository did not provide a complete negative-sampling script, negative samples were generated following the original paper, with 1,000 negative samples constructed for the enzyme side and 1,000 negative samples constructed for the reaction side during training. To improve data-loading efficiency on the present benchmark, sequence and reaction SMILES storage keys were replaced with unique identifiers. The model was retrained on the benchmark using a single NVIDIA RTX 4090 GPU.

CREEP was reproduced from the CARE benchmark implementation (`https://github.com/jsunn-y/CARE`). The original tri-modal enzyme-reaction-text alignment architecture was adapted by removing the text encoder and using the dot product between enzyme and reaction embeddings as the retrieval score. To keep the protein encoder consistent across baselines, the original ProtT5 encoder was replaced with ESM-2 (650M). The model was trained with the BM-NCE loss and otherwise retained the default hyperparameter settings from the released implementation. Retraining was performed using four NVIDIA RTX 4090 GPUs.

GERO-Sep was implemented as a two-stage variant of GERO with the same architecture and inputs. The model was first trained for 100 epochs using retrieval losses only. All parameters except those of the pH and temperature prediction heads were then frozen, and these heads were trained for an additional 70 epochs using the available condition annotations. Thus, both GERO and GERO-Sep received 100 epochs of retrieval training and 70 epochs of condition supervision, differing in whether condition supervision updated the shared representations.

Seq2pH/Topt was used as the enzyme-level catalytic optima predictor in the two-stage baselines. The model predicts optimal pH and temperature from amino acid sequence inputs using protein language-model features and residual prediction heads. For the present comparison, Seq2pH/Topt was reproduced from the open-source implementation (`https://github.com/SizheQiu/Seq2Topt`) and retrained on the present benchmark using a single NVIDIA RTX 4090 GPU. To assess the contribution of GERO's condition predictions with retrieval rankings held fixed, GERO* replaced its pH and temperature estimates with Seq2pH/Topt outputs.

S4. Training and implementation settings

GERO was implemented in PyTorch and trained with AdamW. ESM-2 (650M) and Uni-Mol were used as the protein and molecular encoders, respectively. Their parameters were frozen during training to preserve pre-trained representations and reduce computational cost. AlphaFoldDB version 6 provided predicted structures, and P2Rank version 2.5.1 was used for pocket identification. Only the downstream geometry-aware graph network, projection layers, feature-gating module, and task-specific heads were updated.

For each enzyme, the ESM-2 sequence embedding was obtained by mean pooling the final-layer residue embeddings, excluding special tokens and padding positions. The original ESM embedding dimension was 1280. For the pocket branch, the top-ranked P2Rank pocket was used as the pocket center. Residues with C_α atoms within 15 Å of this center were selected as graph nodes. Each node included an amino acid identity feature and local orientation vectors from C_α to the backbone N atom, backbone C atom, and C_β atom. For glycine, the C_β direction was approximated using the normalized cross product of backbone direction vectors. Directed edges were built with a k-nearest-neighbor graph using $k = 20$, based on C_α distances. Each edge carried the relative displacement vector between the connected residues.

The pocket graph was encoded with a Geometric Vector Perceptron graph neural network. Amino acid identities were mapped to learnable scalar embeddings, and vector features were initialized from the local orientation vectors. A preliminary GVP layer first aligned scalar and vector dimensions, followed by a six-layer GVP graph convolutional network with residual connections and dropout. The final scalar node states were normalized, passed through a readout multilayer perceptron, and mean pooled to produce the pocket embedding. This pocket embedding was concatenated with the projected ESM sequence embedding and then linearly projected to form the enzyme representation.

For each reaction, all available reactants and products were encoded with Uni-Mol and combined by order-invariant mean pooling. The original Uni-Mol reaction embedding dimension was 512. The pooled reaction embedding was projected into the fusion dimension and processed by two pre-norm residual feed-forward blocks. Each block contained layer normalization, a SiLU-activated feed-forward network, and a residual connection. The final reaction representation was layer-normalized before fusion. Both enzyme and reaction representations were mapped to a shared 256-dimensional latent space.

The feature-gating module generated modality-specific gates from the concatenated enzyme and reaction representations. It also computed interaction features using the element-wise product of the two representations. For each modality, the gated residual update combined the original representation, the gate-modulated representation, and the interaction feature, followed by layer normalization. The updated enzyme and reaction representations were then concatenated as the joint pair representation. Three independent two-layer prediction heads with ReLU activation were used to predict the matching logit, optimal pH, and optimal temperature.

Hard-negative mining was performed within each minibatch in both retrieval directions. For each enzyme anchor e_i, cosine similarities were computed between $\mathbf{z}_{e_i}$ and all reaction embeddings in the minibatch. True positives, identical pairs, and candidate pairs with cosine similarity greater than 0.95 were excluded to reduce the risk of selecting false negatives. From the remaining candidates, the top-K most similar reactions were selected as $\mathcal{H}_r^{(i)}$, with $K = 10$. The same filtering and selection procedure was applied to each reaction anchor r_i to obtain the hard negative enzyme set $\mathcal{H}_e^{(i)}$.

The model was trained for 100 epochs with a batch size of 256 on a single NVIDIA RTX 4090 GPU. The base learning rate was 6×10^{-4}, and weight decay was 1×10^{-5}. Training used a 20-epoch linear warm-up from a factor of 0.01, followed by cosine annealing to a minimum learning rate of 1×10^{-6}. Gradients were clipped at a maximum norm of 1.0. The loss weights were $\lambda_{\text{hard}} = 10.0$ and $\lambda_{\text{cond}} = 0.75$. Condition-regression loss was introduced at epoch 30, allowing the model to first learn enzyme-reaction alignment before optimizing pH and temperature prediction.

Redox-coenzyme sensitivity was evaluated at inference using the seed-42 models. Eight coenzymes-NAD^+, NADH, $NADP^+$, NADPH, FMN, $FMNH_2$, FAD, and $FADH_2$-were identified by exact canonical isomeric SMILES matching. Reaction embeddings were recomputed with these participants retained or excluded using the same molecular encoder, while model weights and candidate pools were held fixed. Exclusion modified 1,018 of 7,572 reactions and removed 1,986 participant entries. One additional reaction retained its original representation because exclusion would otherwise remove all participants. No reaction records were discarded.

S5. Ecological-niche analysis of catalytic-condition predictions

To assess whether GERO-predicted catalytic conditions are consistent with the environmental adaptation of source organisms, we independently annotated ecological niches for enzymes in the Enzyme-SIM test set. Temperature niches were obtained from TEMPURA, TOMER/Seq2Topt, and BacDive and classified as psychrophilic ($T < 20\,^\circ$C), mesophilic ($20\,^\circ\text{C} \leq T < 45\,^\circ$C), or thermophilic ($T \geq 45\,^\circ$C). pH niches were derived from BacDive optimal-growth-pH or phenotypic records and classified as acidophilic (pH < 5.0), neutrophilic (pH 5.0-8.5), or alkaliphilic (pH > 8.5). Records lacking explicit supporting evidence were excluded.

For each enzyme, one known positive reaction was randomly selected using a fixed seed as the prediction context. Predicted temperatures in the mesophilic and thermophilic groups were compared using a two-sided Mann-Whitney U test. A 95% confidence interval for the between-group mean difference was estimated from 5,000 enzyme-level bootstrap replicates. Robustness to reaction-context selection was assessed through 1,000 repeated random samplings. Observed and predicted values were also compared across all 225 test enzymes to assess prediction errors across the full label range (Figure S4B and D).

Temperature-niche annotations covered 62 enzymes (1 psychrophilic, 45 mesophilic, and 16 thermophilic), including 47 supported by exact strain, taxon, or protein-accession matches. Mean predicted optimal temperatures were 33.76 °C and 58.37 °C for the mesophilic and thermophilic groups, respectively, with a mean difference of 24.60 °C (bootstrap 95% CI: 17.16-32.63 °C; Mann-Whitney $U = 35$, $p = 1.04 \times 10^{-7}$; absolute rank-biserial effect size = 0.903). This difference remained stable across reaction-context resamplings. pH-niche annotations covered only two acidophilic and three neutrophilic enzymes, with no alkaliphilic samples. Their mean predicted optimal pH values were 6.68 and 8.14, respectively; these comparisons were interpreted descriptively because of the small sample sizes (Figure S4A and C).

S6. Context controls for catalytic-condition prediction

The experiment included a training-mean predictor, four condition-prediction controls, and jointly trained GERO. The mean predictor outputs the pH and temperature means calculated separately over unique training

enzymes with the corresponding labels (Table S1). The four controls used enzyme-only input, molecular reaction context, complete four-level EC encoding, and hierarchical EC encoding. Enzyme-only input used a zero reaction vector. Molecular reaction context used 512-dimensional Uni-Mol reaction features projected to 256 dimensions. The EC controls represented complete four-level categories or prefixes from levels one through four as normalized multi-hot vectors, followed by projection to 256 dimensions. EC vocabularies were constructed from condition-labelled training reactions. Unseen categories were omitted at test time, and inputs without known categories were represented by zero vectors.

Within each seed, the four controls shared the same frozen GERO-Sep enzyme encoder trained solely with retrieval supervision, identical initial weights for the fusion layer and condition-prediction heads, and the same training samples and batch order. The condition heads used a $512 \rightarrow 256 \rightarrow 1$ architecture. Training minimized MSE on standardized condition labels, with missing labels masked and no retrieval loss. All controls were trained using AdamW for 70 epochs with a batch size of 256, weight decay of 10^{-5}, and cosine learning-rate decay from 6×10^{-4} to 10^{-6}. Final-epoch models were evaluated. Jointly trained GERO used the main-experiment checkpoints.

Condition evaluation excluded enzymes with either pH or temperature annotated in the corresponding training partition. MAE and RMSE were calculated separately over unique test enzymes with the corresponding labels. One known positive reaction per test enzyme was randomly selected using a fixed sampling seed and held constant across GERO, the context-based controls, and all model seeds. Enzyme-only and mean predictors did not use reaction context.

For Hit@k-TR, all methods shared GERO's retrieval rankings and full candidate pools within each seed, using their own condition predictions for candidate enzyme-reaction pairs to calculate hit rates. The tolerances were ± 0.5 pH units and $\pm 5\,°\mathrm{C}$. Evaluation required both condition labels, yielding 225 E$\rightarrow$R and 416 R$\rightarrow$E queries on Enzyme-SIM, and 131 and 106, respectively, on Reaction-SIM. Tables S6 and S7 report means and sample standard deviations across five seeds (40-44). The mean predictor's regression metrics are invariant across seeds, whereas its Hit@k-TR varies with retrieval rankings.

Supplementary figures

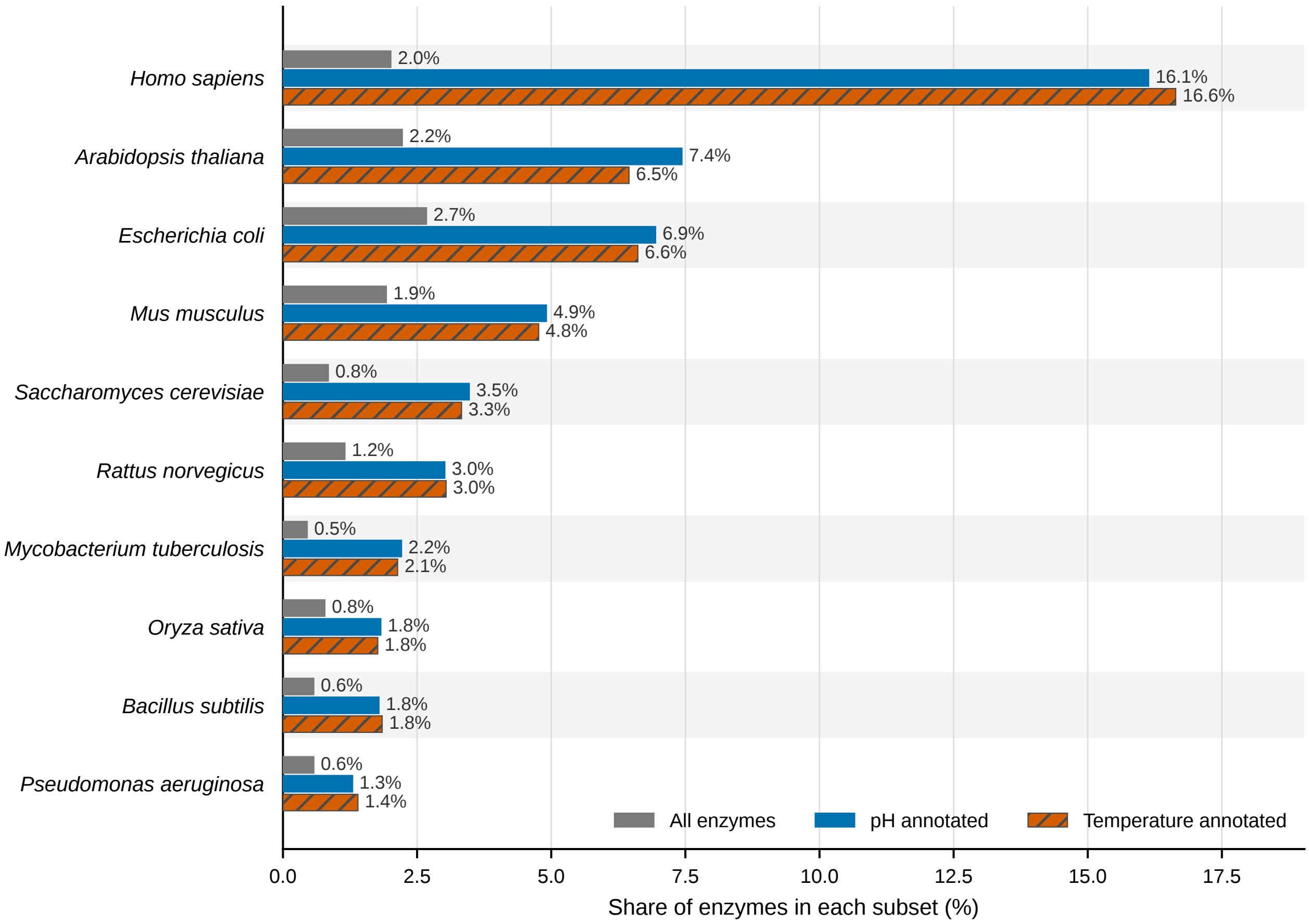

Figure S1: Species composition of enzymes with pH and temperature annotations. Gray, blue, and orange bars show the percentage of all enzymes, pH-annotated enzymes, and temperature-annotated enzymes, respectively, contributed by each of the 10 most frequently condition-annotated species.

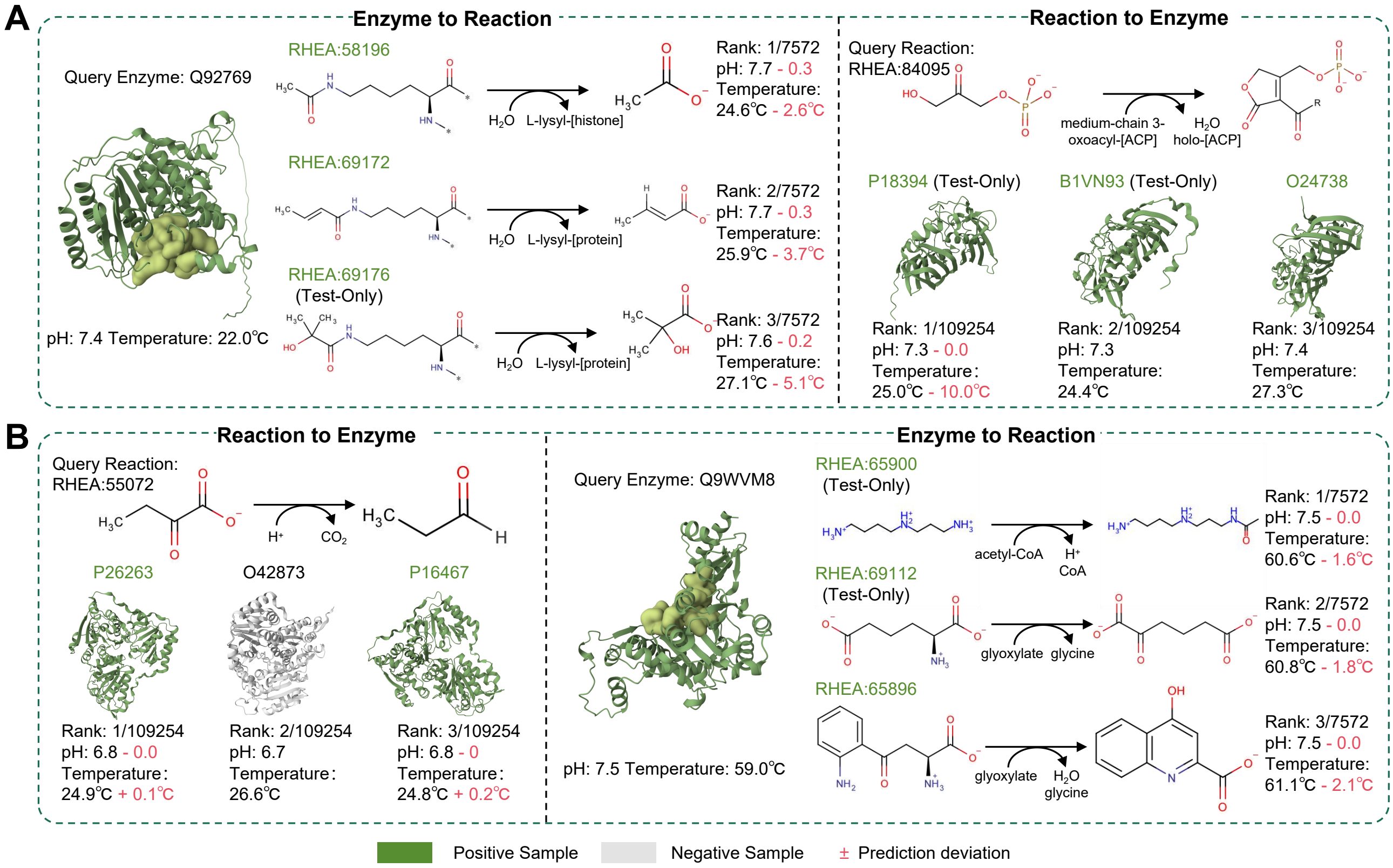

Figure S2: Illustrative benchmark retrieval examples. A, Top-ranked candidate reactions for query enzyme Q92769 and top-ranked candidate enzymes for query reaction RHEA:84095 on Enzyme-SIM. B, Top-ranked candidate enzymes for query reaction RHEA:55072 and top-ranked candidate reactions for query enzyme Q9WVM8 on Reaction-SIM. Predicted pH and temperature values are shown for each retrieved candidate, with deviations from curated enzyme-level annotations indicated when available. Note: Q9WVM8 appeared in the training set. These illustrative examples use the full benchmark libraries of 7,572 reactions and 109,254 enzymes; candidate pools for the main benchmark are specified separately in Table S2.

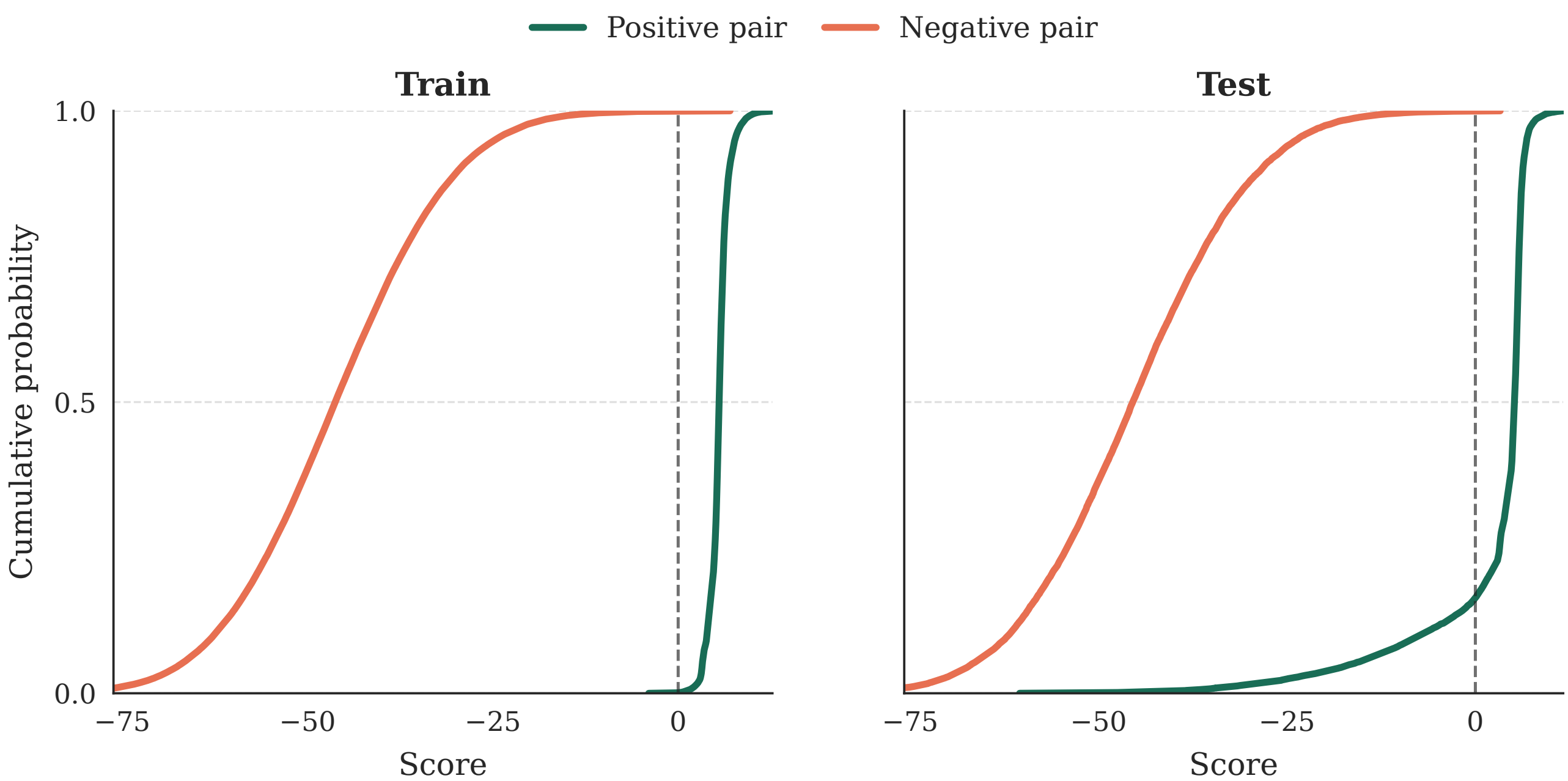

Figure S3: Alignment-score distributions for positive and random negative enzyme-reaction pairs in the training and test sets.

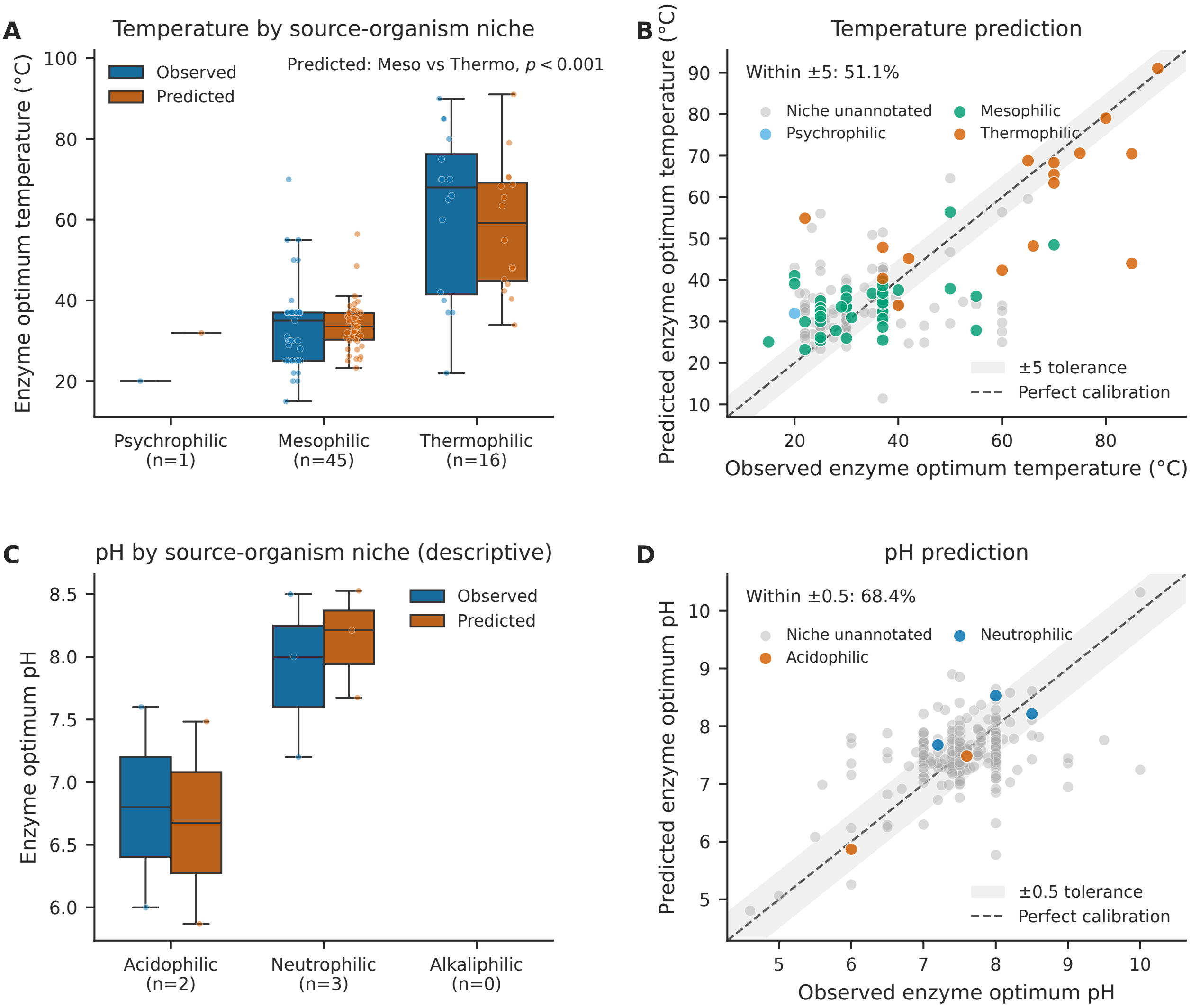

Figure S4: Ecological-niche stratification and catalytic-condition predictions on Enzyme-SIM. (A, C) Observed and predicted optimal temperatures and pH values grouped by source-organism niche, with group sizes indicated. (B, D) Observed versus predicted values for all 225 test enzymes. Gray points indicate enzymes without niche annotations; dashed lines indicate perfect agreement, and shaded bands indicate tolerances of ±5 °C and ±0.5 pH units, respectively.

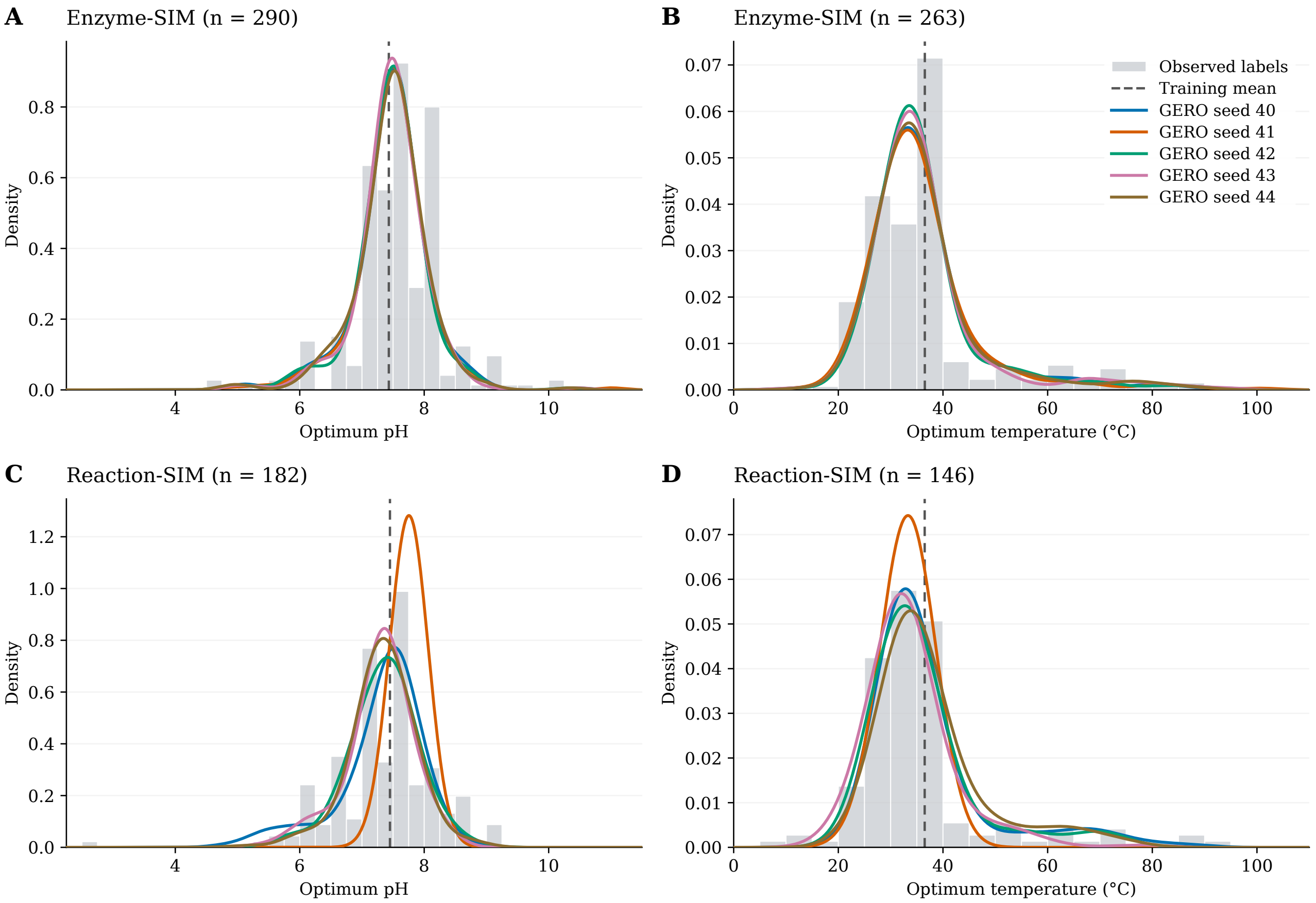

Figure S5: Observed and predicted pH (A, C) and temperature (B, D) distributions on Enzyme-SIM (A, B) and Reaction-SIM (C, D). Gray histograms show observed labels, colored curves show GERO predictions across five seeds, and dashed lines indicate training-set means. Predictions use each seed's retrieved Top-1 reaction as context.

Supplementary tables

Table S1: Dataset composition, enzyme-level condition-annotation coverage, and condition-label distributions for the Enzyme-SIM and Reaction-SIM benchmark splits.

	Enzyme-SIM			Reaction-SIM		
**Category**	**Train**	**Test**	**Total**	**Train**	**Test**	**Total**
Enzyme-reaction pairs	136,658	15,721	152,379	142,204	10,175	152,379
Unique enzymes	98,344	10,910	109,254	102,908	7,803	109,254
Unique reactions	7,124	1,768	7,572	6,928	644	7,572
Enzymes with pH annotations	2,561 (2.60%)	290 (2.66%)	2,851 (2.61%)	2,669 (2.59%)	304 (3.90%)	2,851 (2.61%)
Enzymes with temperature annotations	2,171 (2.21%)	263 (2.41%)	2,434 (2.23%)	2,288 (2.22%)	252 (3.23%)	2,434 (2.23%)
Enzymes with both annotations	1,854 (1.89%)	225 (2.06%)	2,079 (1.90%)	1,948 (1.89%)	212 (2.72%)	2,079 (1.90%)
pH, mean ± SD	7.43 ± 0.86	7.50 ± 0.72	-	7.45 ± 0.85	7.28 ± 0.81	-
Temperature (°C), mean ± SD	36.53 ± 14.34	35.14 ± 12.44	-	36.50 ± 14.18	34.43 ± 13.51	-

Counts and annotation percentages refer to the original subsets before condition-evaluation filtering. Percentages are calculated relative to the number of unique enzymes in each subset. Enzyme-SIM partitions enzyme-sequence clusters, with no shared enzymes but 1,320 shared reactions between training and test sets. Reaction-SIM partitions reaction-fingerprint clusters, with no shared reactions but 1,457 shared enzymes, including 122 with pH annotations, 106 with temperature annotations, and 81 with both. For Reaction-SIM, test-set condition-label means and standard deviations are calculated only over unique annotated enzymes absent from the training set.

Table S2: Numbers of test queries and candidates for bidirectional retrieval.

**Dataset**	**Direction**	**MRR / Hit@k**		**Hit@k-TR**	
		**Queries**	**Candidates**	**Queries**	**Candidates**
Enzyme-SIM	E→R	10,910	7,572	225	7,572
	R→E	1,768	10,910	416	10,910
Reaction-SIM	E→R	7,803	644	131	644
	R→E	644	109,254	106	109,254

Table S3: Hit@k-TR performance under different pH and temperature tolerance thresholds on Enzyme-SIM. Strict, Primary, and Relaxed correspond to $(\Delta\text{pH}, \Delta T) = (0.25, 2.5\,^\circ\text{C})$, $(0.5, 5\,^\circ\text{C})$, and $(1.0, 10\,^\circ\text{C})$, respectively. Values are reported as mean ± sample standard deviation (%).

**Enzyme-SIM**	**Methods**	**Hit@1-TR-Strict**	**Hit@10-TR-Strict**	**Hit@1-TR-Primary**	**Hit@10-TR-Primary**	**Hit@1-TR-Relaxed**	**Hit@10-TR-Relaxed**
E→R	SimSearch	4.1778 ± 0.5963	5.2444 ± 0.9635	17.1556 ± 1.3184	21.0667 ± 1.4606	**42.2222 ± 0.8889**	51.1111 ± 1.4402
	CREEP	3.5556 ± 0.8889	5.1556 ± 1.0232	13.6000 ± 1.2008	20.5333 ± 1.9727	33.9556 ± 2.0988	51.7333 ± 2.3851
	ReactZyme	0.0000 ± 0.0000	0.4444 ± 0.3143	0.3556 ± 0.1988	2.4000 ± 1.1590	0.3556 ± 0.1988	5.2444 ± 2.6181
	GERO*	3.7333 ± 0.6740	5.3333 ± 0.8889	15.5556 ± 1.0423	21.8667 ± 1.3842	37.7778 ± 1.8325	53.4222 ± 1.5839
	GERO-Sep	7.0222 ± 1.1504	11.8222 ± 0.6740	16.0000 ± 0.8889	24.8889 ± 1.3333	32.7111 ± 3.2750	46.3111 ± 3.3614
	GERO	**8.9778 ± 1.6147**	**13.3333 ± 1.6330**	**22.4000 ± 2.1222**	**32.5333 ± 2.6925**	39.7333 ± 2.8906	**57.0667 ± 1.9525**
R→E	CREEP	1.0577 ± 0.6925	6.3942 ± 1.5448	4.3750 ± 1.4262	23.5096 ± 2.2512	9.8077 ± 1.3210	**51.4423 ± 2.6112**
	ReactZyme	0.0000 ± 0.0000	0.0000 ± 0.0000	0.0000 ± 0.0000	0.0962 ± 0.1317	0.0000 ± 0.0000	0.2404 ± 0.2944
	GERO*	1.3942 ± 0.9969	5.8173 ± 1.9852	4.8558 ± 1.5146	20.4808 ± 1.2878	11.6827 ± 1.6878	45.9135 ± 1.9895
	GERO-Sep	3.4135 ± 0.8222	11.2981 ± 2.5269	7.5000 ± 1.1198	23.9904 ± 2.5968	14.5673 ± 2.1066	42.4038 ± 3.1046
	GERO	**4.0865 ± 0.4808**	**18.9423 ± 3.1909**	**9.8077 ± 0.7090**	**34.3269 ± 1.4160**	**14.7596 ± 0.9998**	49.9038 ± 1.2900

Table S4: Hit@k-TR performance under different pH and temperature tolerance thresholds on Reaction-SIM. Strict, Primary, and Relaxed correspond to $(\Delta\text{pH}, \Delta T) = (0.25, 2.5\,°\text{C})$, $(0.5, 5\,°\text{C})$, and $(1.0, 10\,°\text{C})$, respectively. Values are reported as mean ± sample standard deviation (%).

Reaction-SIM	Methods	Hit@1-TR-Strict	Hit@10-TR-Strict	Hit@1-TR-Primary	Hit@10-TR-Primary	Hit@1-TR-Relaxed	Hit@10-TR-Relaxed
E→R	CREEP	0.9160 ± 1.2543	**3.6641 ± 1.3655**	2.4427 ± 0.9953	10.3817 ± 2.3279	**9.3130 ± 0.9953**	**27.6336 ± 4.6870**
	ReactZyme	0.0000 ± 0.0000	0.4580 ± 0.6828	0.0000 ± 0.0000	1.0687 ± 1.0242	0.0000 ± 0.0000	4.4275 ± 1.9759
	GERO*	0.3053 ± 0.4181	2.2901 ± 1.5267	1.8321 ± 1.7574	8.8550 ± 1.2773	7.0229 ± 1.3655	22.4427 ± 3.7241
	GERO-Sep	**1.0687 ± 0.8704**	1.2214 ± 1.1577	1.8321 ± 1.6724	4.1221 ± 2.7839	4.5802 ± 2.2256	10.3817 ± 3.8396
	GERO	0.7634 ± 0.9349	2.1374 ± 1.2543	**2.7481 ± 1.8384**	**10.5344 ± 3.0725**	4.5802 ± 1.4281	19.0840 ± 2.4736
R→E	SimSearch	0.0000 ± 0.0000	0.0000 ± 0.0000	0.0000 ± 0.0000	0.0000 ± 0.0000	0.0000 ± 0.0000	0.0000 ± 0.0000
	CREEP	0.0000 ± 0.0000	0.0000 ± 0.0000	0.0000 ± 0.0000	0.3774 ± 0.8438	0.0000 ± 0.0000	**2.2642 ± 2.1719**
	ReactZyme	0.0000 ± 0.0000	0.0000 ± 0.0000	0.0000 ± 0.0000	0.0000 ± 0.0000	0.0000 ± 0.0000	0.0000 ± 0.0000
	GERO*	0.0000 ± 0.0000	**0.3774 ± 0.5167**	**0.3774 ± 0.8438**	1.3208 ± 0.8438	**0.5660 ± 0.8438**	**2.2642 ± 1.5786**
	GERO-Sep	0.0000 ± 0.0000	0.1887 ± 0.4219	0.1887 ± 0.4219	0.7547 ± 0.7893	0.3774 ± 0.5167	1.6981 ± 0.7893
	GERO	0.0000 ± 0.0000	**0.3774 ± 0.5167**	**0.3774 ± 0.8438**	**1.6981 ± 0.7893**	**0.5660 ± 0.8438**	**2.2642 ± 1.5786**

Table S5: Bidirectional enzyme-reaction retrieval performance on the same evaluation subset used for Hit@k-TR under the Enzyme-SIM and Reaction-SIM splits. Best results in each column are highlighted in bold, including ties.

	Enzyme-SIM/E→R		Enzyme-SIM/R→E		Reaction-SIM/E→R		Reaction-SIM/R→E	
Methods	Hit@1	Hit@10	Hit@1	Hit@10	Hit@1	Hit@10	Hit@1	Hit@10
SimSearch	**0.6178**	0.7467	-	-	-	-	**0.0377**	0.0377
CREEP	0.5129 ± 0.0210	0.7760 ± 0.0120	0.5399 ± 0.0403	**0.8101 ± 0.0149**	**0.1359 ± 0.0100**	**0.4321 ± 0.0544**	0.0321 ± 0.0143	0.1038 ± 0.0447
ReactZyme	0.0053 ± 0.0049	0.0676 ± 0.0389	0.0192 ± 0.0163	0.0389 ± 0.0291	0.0000 ± 0.0000	0.0504 ± 0.0251	0.0000 ± 0.0000	0.0000 ± 0.0000
GERO*	0.5698 ± 0.0207	0.7849 ± 0.0107	0.5457 ± 0.0124	0.7731 ± 0.0163	0.1099 ± 0.0184	0.3344 ± 0.0312	**0.0377 ± 0.0149**	0.1075 ± 0.0196
GERO-Sep	0.5884 ± 0.0188	**0.7867 ± 0.0122**	**0.5654 ± 0.0281**	0.7808 ± 0.0129	0.1313 ± 0.0250	0.3420 ± 0.0267	**0.0377 ± 0.0241**	**0.1358 ± 0.0143**
GERO	0.5698 ± 0.0207	0.7849 ± 0.0107	0.5457 ± 0.0124	0.7731 ± 0.0163	0.1099 ± 0.0184	0.3344 ± 0.0312	**0.0377 ± 0.0149**	0.1075 ± 0.0196

Table S6: Condition-prediction performance on Enzyme-SIM. Values are mean ± sample SD (%) across five seeds (40-44). The best and second-best results are shown in bold and underlined, respectively, based on their mean values.

Method	pH MAE ↓	pH RMSE ↓	Temperature MAE ↓	Temperature RMSE ↓	E→R Hit@1-TR ↑	E→R Hit@10-TR ↑	R→E Hit@1-TR ↑	R→E Hit@10-TR ↑
Training mean	0.5020	0.7219	8.4525	12.4964	15.91 ± 1.38	21.78 ± 0.54	4.66 ± 1.06	21.83 ± 1.82
GERO	**0.4965 ± 0.0118**	**0.6843 ± 0.0120**	**7.1765 ± 0.2215**	**10.2611 ± 0.2250**	**22.40 ± 2.12**	**32.53 ± 2.69**	**9.81 ± 0.71**	**34.33 ± 1.42**
Enzyme-only	0.6198 ± 0.0229	0.8186 ± 0.0353	9.1236 ± 0.3907	12.3104 ± 0.5673	15.29 ± 1.02	20.71 ± 0.81	6.39 ± 1.27	24.42 ± 4.28
EC4 context	0.5726 ± 0.0170	0.7743 ± 0.0212	8.6270 ± 0.2583	12.2291 ± 0.4826	17.16 ± 1.49	25.87 ± 2.00	7.50 ± 1.90	31.39 ± 2.54
Hierarchical EC	0.5353 ± 0.0112	0.7218 ± 0.0116	8.3995 ± 0.3563	11.8988 ± 0.6262	17.78 ± 1.63	27.82 ± 1.56	8.65 ± 1.67	32.02 ± 4.07
Reaction context	0.5859 ± 0.0272	0.7791 ± 0.0308	8.4259 ± 0.1869	11.6986 ± 0.5916	17.33 ± 2.18	25.42 ± 2.76	7.84 ± 1.92	29.18 ± 4.05

Table S7: Condition-prediction performance on Reaction-SIM. Values are mean ± sample SD (%) across five seeds (40-44). The best and second-best results are shown in bold and underlined, respectively, based on their mean values.

Method	pH MAE ↓	pH RMSE ↓	Temperature MAE ↓	Temperature RMSE ↓	E→R Hit@1-TR ↑	E→R Hit@10-TR ↑	R→E Hit@1-TR ↑	R→E Hit@10-TR ↑
Training mean	**0.5865**	0.8255	**9.2919**	**13.6239**	2.60 ± 1.28	6.11 ± 0.93	0.00 ± 0.00	0.38 ± 0.52
GERO	0.6133 ± 0.0535	0.8563 ± 0.0542	9.3142 ± 0.4407	14.1745 ± 0.7612	**2.75 ± 1.84**	**10.53 ± 3.07**	**0.38 ± 0.84**	**1.70 ± 0.79**
Enzyme-only	0.6979 ± 0.0530	0.9113 ± 0.0679	11.2365 ± 1.0428	15.8074 ± 1.1864	1.22 ± 1.02	5.34 ± 3.28	**0.38 ± 0.84**	1.51 ± 1.08
EC4 context	0.6309 ± 0.0289	0.8411 ± 0.0224	10.0447 ± 0.2908	14.1011 ± 0.4320	1.83 ± 0.68	7.02 ± 2.25	0.00 ± 0.00	0.57 ± 0.84
Hierarchical EC	0.5976 ± 0.0310	**0.7988 ± 0.0263**	9.8842 ± 0.4479	14.1887 ± 0.6140	1.07 ± 0.68	6.56 ± 1.39	**0.38 ± 0.84**	**1.70 ± 1.03**
Reaction context	0.6367 ± 0.0283	0.8485 ± 0.0392	10.9317 ± 0.4522	15.4468 ± 0.3414	1.37 ± 1.25	5.65 ± 2.62	**0.38 ± 0.84**	1.13 ± 0.79

Table S8: Seed-42 ablation results for Enzyme-SIM retrieval, comparing pocket, fusion, and reaction-encoder variants. Bold values indicate the highest score for each metric within each retrieval direction, including ties.

Enzyme-SIM	Methods	MRR	Hit@1	Hit@10	Hit@1-TR	Hit@10-TR
E→R	w/oPocket	0.8983	0.8720	0.9411	0.1911	0.3111
	w/oFusion	0.8727	0.8405	0.9286	0.1244	0.2089
	RxnFP	0.8970	0.8670	**0.9439**	0.1911	0.2844
	ChemBERTa	0.8903	0.8568	0.9404	0.2133	0.3111
	GERO	**0.9032**	**0.8785**	0.9419	**0.2356**	**0.3556**
R→E	w/oPocket	**0.6422**	**0.5781**	**0.7777**	0.0769	0.3486
	w/oFusion	0.6044	0.5379	0.7381	0.0649	0.3029
	RxnFP	0.6396	0.5713	0.7692	**0.1010**	0.3822
	ChemBERTa	0.6307	0.5650	0.7636	0.0962	**0.3894**
	GERO	0.6398	0.5718	0.7726	0.0938	0.3221

Table S9: Seed-42 ablation results for Reaction-SIM retrieval, comparing pocket, fusion, and reaction-encoder variants. Bold values indicate the highest score for each metric within each retrieval direction, including ties.

Reaction-SIM	Methods	MRR	Hit@1	Hit@10	Hit@1-TR	Hit@10-TR
E→R	w/oPocket	0.2445	0.1597	0.3782	**0.0229**	**0.0992**
	w/oFusion	0.2302	0.1471	0.4091	**0.0229**	0.0687
	RxnFP	0.2613	0.2045	0.3587	**0.0229**	0.0687
	ChemBERTa	0.2603	0.1901	0.3959	0.0000	0.0763
	GERO	**0.2992**	**0.2077**	**0.4858**	**0.0229**	0.0687
R→E	w/oPocket	0.1852	0.1413	0.2624	0.0000	0.0094
	w/oFusion	0.1543	0.1087	0.2391	0.0000	**0.0189**
	RxnFP	**0.2127**	**0.1708**	**0.2873**	**0.0000**	0.0094
	ChemBERTa	0.1736	0.1304	0.2422	0.0000	0.0000
	GERO	0.1910	0.1444	0.2764	0.0000	0.0094

Table S10: Effect of P2Rank pocket selection on retrieval performance. Top-1 and Lowest denote the highest- and lowest-scoring valid pockets; Δ denotes Top-1 minus Lowest. Model weights, query sets, and candidate pools were fixed between conditions. E→R used test enzymes with at least two valid pockets, ranking 7,572 and 644 reaction candidates on Enzyme-SIM and Reaction-SIM, respectively. R→E used all test reactions and 109,254 enzyme candidates; only eligible test enzymes underwent pocket replacement, while all other candidates retained their original Top-1 pockets. n denotes the number of queries.

Dataset	Direction	n	Metric	Top-1	Lowest	Δ
Enzyme-SIM	E→R	9,734	MRR	0.8999	0.7770	+0.1229
			Hit@1	0.8758	0.7392	+0.1366
			Hit@10	0.9381	0.8406	+0.0975
	R→E	1,768	MRR	0.1130	0.0611	+0.0520
			Hit@1	0.0271	0.0113	+0.0158
			Hit@10	0.3196	0.1640	+0.1555
Reaction-SIM	E→R	6,838	MRR	0.2967	0.2168	+0.0799
			Hit@1	0.1982	0.1401	+0.0581
			Hit@10	0.4971	0.3717	+0.1253
	R→E	644	MRR	0.1910	0.1122	+0.0788
			Hit@1	0.1444	0.0714	+0.0730
			Hit@10	0.2764	0.1832	+0.0932

Table S11: GERO benchmark retrieval performance with redox coenzymes retained or excluded at inference. Comparisons use fixed seed-42 models and unchanged candidate pools. Exclusion modified 178 of 1,768 Enzyme-SIM test reactions and 2 of 644 Reaction-SIM test reactions.

**Dataset**	**Direction**	**Coenzyme input**	**MRR**	**Hit@1**	**Hit@10**
Enzyme-SIM	E→R	Retained	0.9037	0.8795	0.9419
		Excluded	0.8760	0.8507	0.9166
	R→E	Retained	0.6398	0.5718	0.7726
		Excluded	0.6016	0.5396	0.7251
Reaction-SIM	E→R	Retained	0.2992	0.2076	0.4860
		Excluded	0.2991	0.2076	0.4857
	R→E	Retained	0.1910	0.1444	0.2764
		Excluded	0.1911	0.1444	0.2764

Table S12: Effects of redox coenzyme inclusion on target-enzyme rankings in the iodinin case study(excluded → retained).

**Step**	**Enzyme**	**GERO**	**ReactZyme**	**CREEP**
1	xpzG	6 → 7	109 → 107	35 → 137
2	xpzG	6 → 5	119 → 108	184 → 139
3	xpzH	84 → 87	12 → 12	56 → 50
3	phzNO1	39 → 68	22 → 22	179 → 53
4	xpzH	93 → 110	12 → 12	23 → 61
4	phzNO1	45 → 70	20 → 23	171 → 86

Table S13: Paired comparison of GERO and GERO-Sep for Hit@10-TR. Δ denotes the mean improvement over GERO-Sep across five seeds, in percentage points. Seed columns report two-sided exact McNemar p-values, Holm-adjusted across 20 comparisons, using tolerances of ±0.5 pH units and ±5 °C. Bold values indicate adjusted $p < 0.05$.

Setting	Δ	Seed 40	Seed 41	Seed 42	Seed 43	Seed 44
Enzyme-SIM/E→R	7.64	0.0726	0.7245	**0.0249**	0.0552	0.9732
Enzyme-SIM/R→E	10.34	$\mathbf{1.69 \times 10^{-5}}$	**0.0066**	$\mathbf{3.65 \times 10^{-4}}$	$\mathbf{3.81 \times 10^{-4}}$	$\mathbf{1.02 \times 10^{-9}}$
Reaction-SIM/E→R	6.41	$\mathbf{4.88 \times 10^{-4}}$	1.0000	1.0000	**0.0137**	1.0000
Reaction-SIM/R→E	0.94	1.0000	1.0000	1.0000	1.0000	1.0000
